# Supplementary figures and images for: Genomics of Preaxostyla Flagellates Illuminates the Path Towards the Loss of Mitochondria
Source: PLoS Genet. 2023 Dec 7;19(12):e1011050. doi: 10.1371/journal.pgen.1011050 (PMC10703272; doi:10.1371/journal.pgen.1011050)

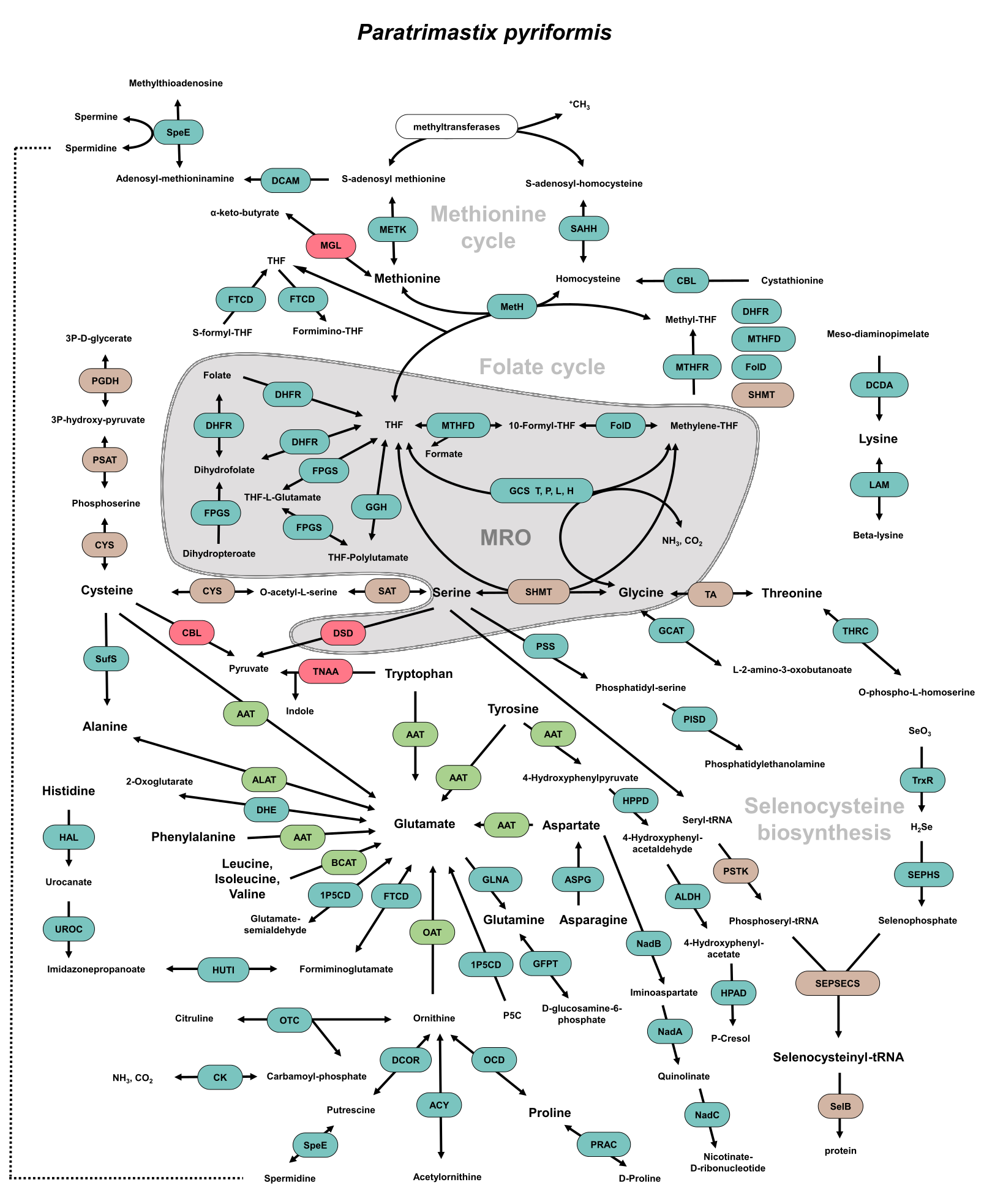

Supplement: S1 Fig — Brown color indicates enzymes possibly involved in amino acid biosynthesis pathways. Red color indicates enzymes possibly involved in ATP production. Note that some of the connections between metabolites correspond to the mere transfer of the amino group rather than conversion of the carbon backbone of the molecule (green color). Cyan color is used for remaining enzymes. Abbreviations and Enzyme Commission numbers are given in S6 File. (TIF) [file pgen.1011050.s001.tif]

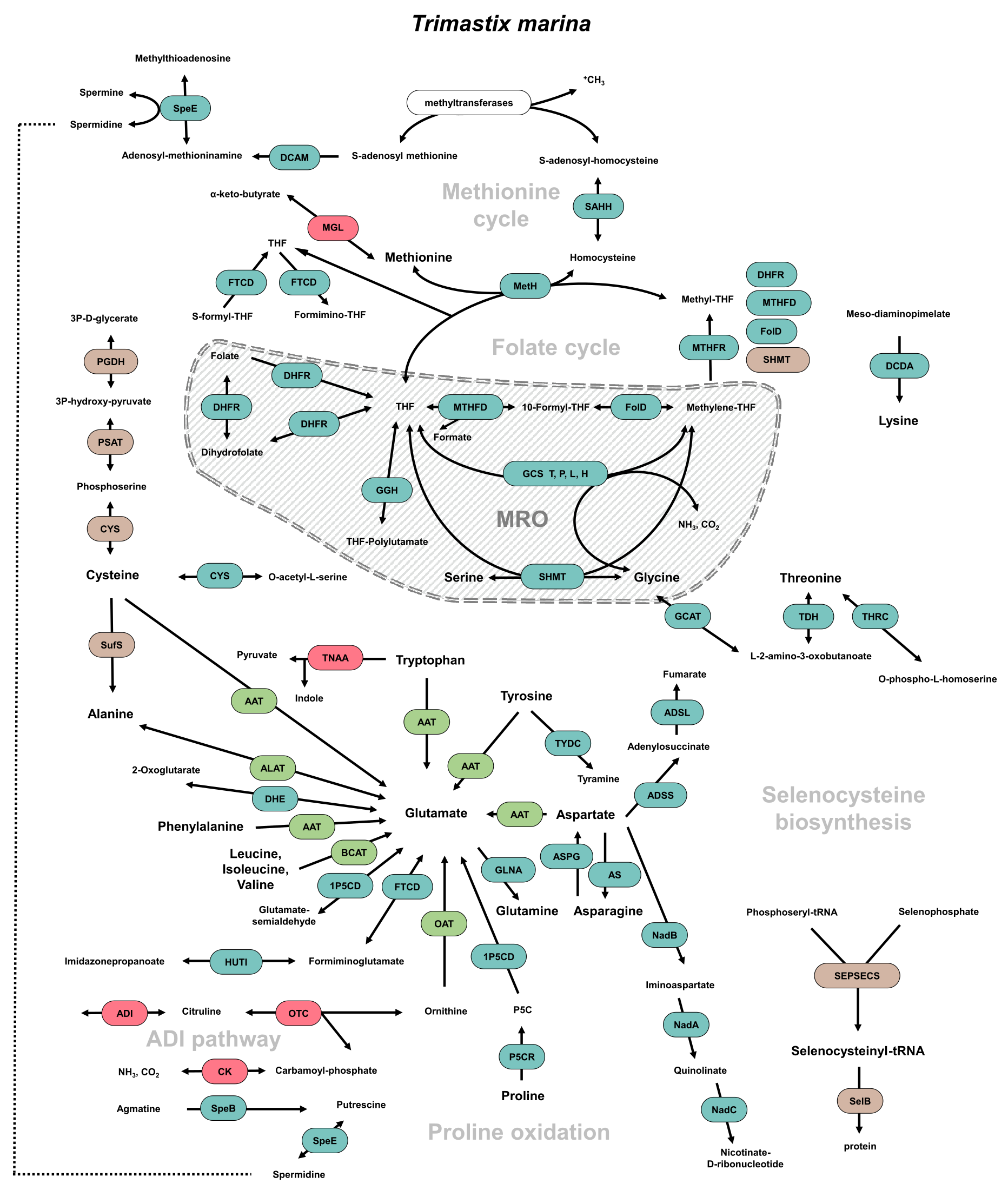

Supplement: S2 Fig — Brown color indicates enzymes possibly involved in amino acid biosynthesis pathways. Red color indicates enzymes possibly involved in ATP production. Note that some of the connections between metabolites correspond to the mere transfer of the amino group rather than conversion of the carbon backbone of the molecule (green color). Cyan color is used for remaining enzymes. Abbreviations and Enzyme Commission numbers are given in S6 File. (TIF) [file pgen.1011050.s002.tif]

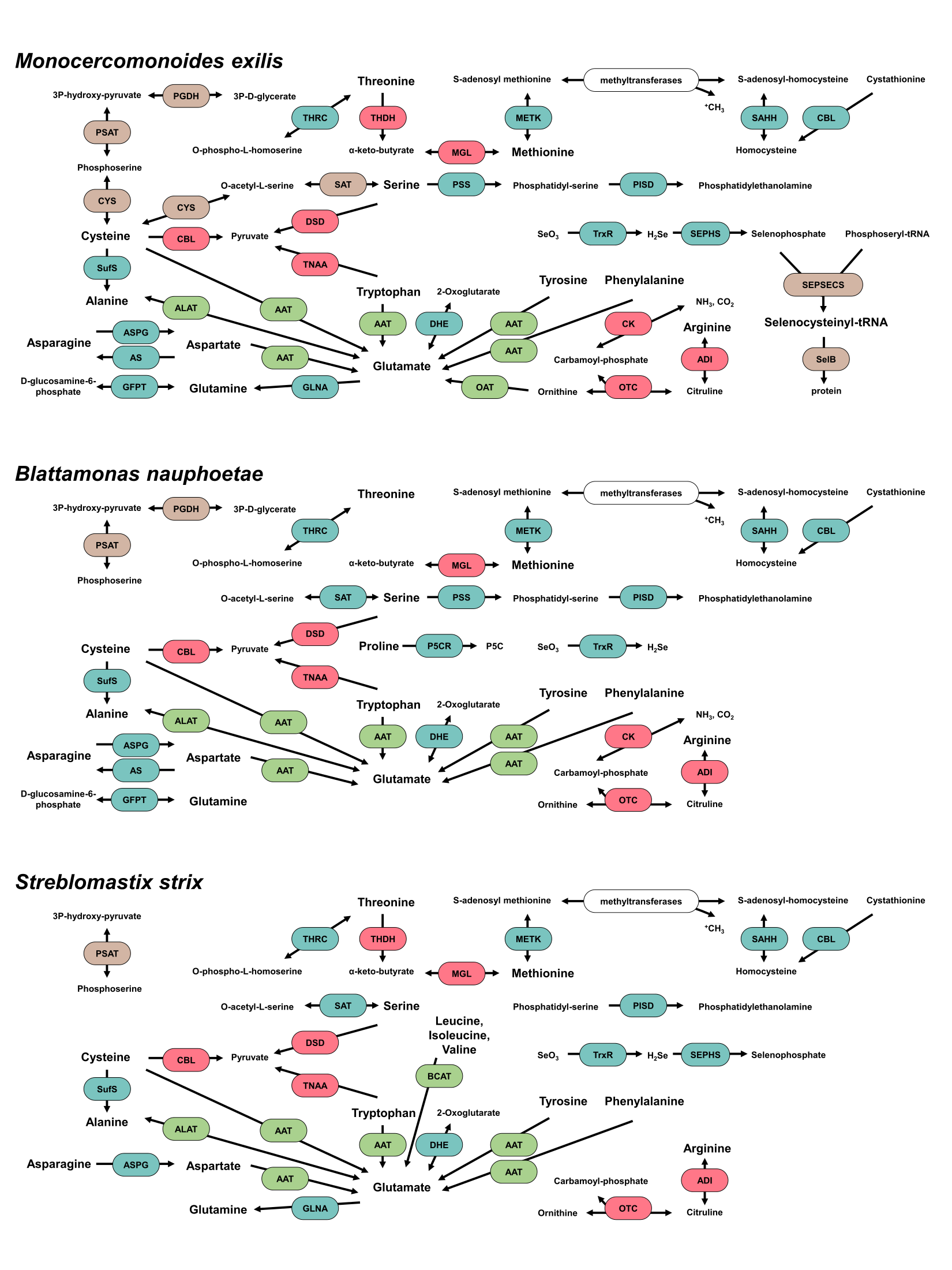

Supplement: S3 Fig — Brown color indicates enzymes possibly involved in amino acid biosynthesis pathways. Red color indicates enzymes possibly involved in ATP production. Note that some of the connections between metabolites correspond to the mere transfer of the amino group rather than conversion of the carbon backbone of the molecule (green color). Cyan color is used for remaining enzymes. Abbreviations and Enzyme Commission numbers are given in S6 File. (TIF) [file pgen.1011050.s003.tif]

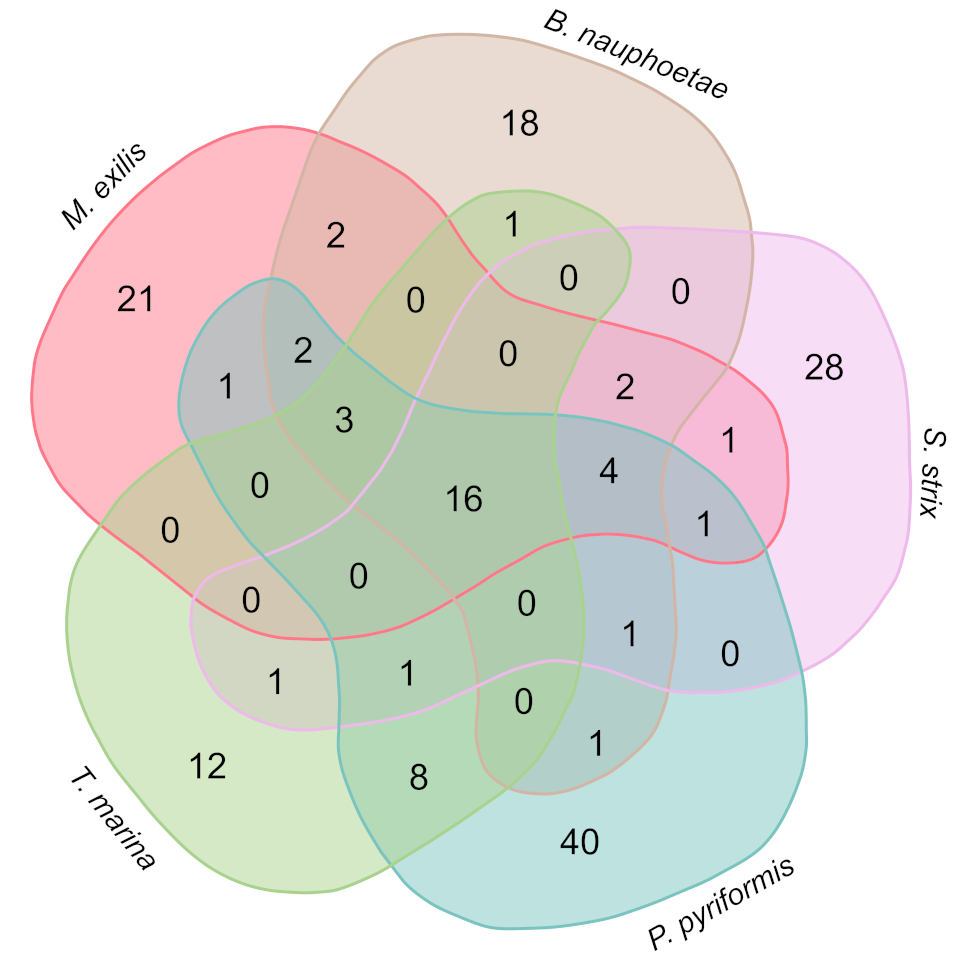

Supplement: S4 Fig — The identity of the OGs and of the component proteins are provided in S7 File. (TIF) [file pgen.1011050.s004.tif]
