# Supplementary material for: Genomics of Preaxostyla Flagellates Illuminates the Path Towards the Loss of Mitochondria
Source: PLoS Genet. 2023 Dec 7;19(12):e1011050. doi: 10.1371/journal.pgen.1011050 (PMC10703272; doi:10.1371/journal.pgen.1011050)

S4 File

A) DLP

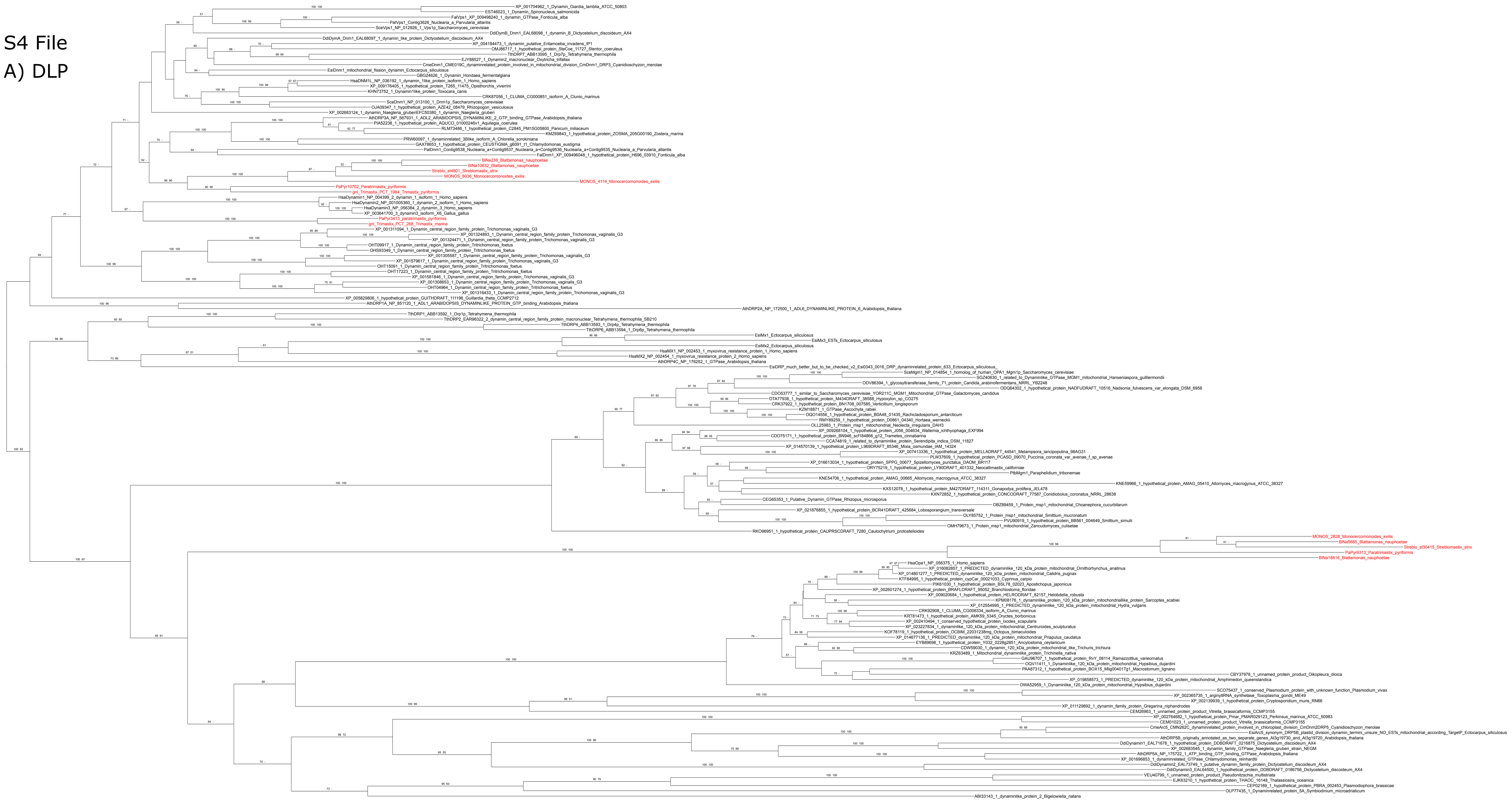

B) ME

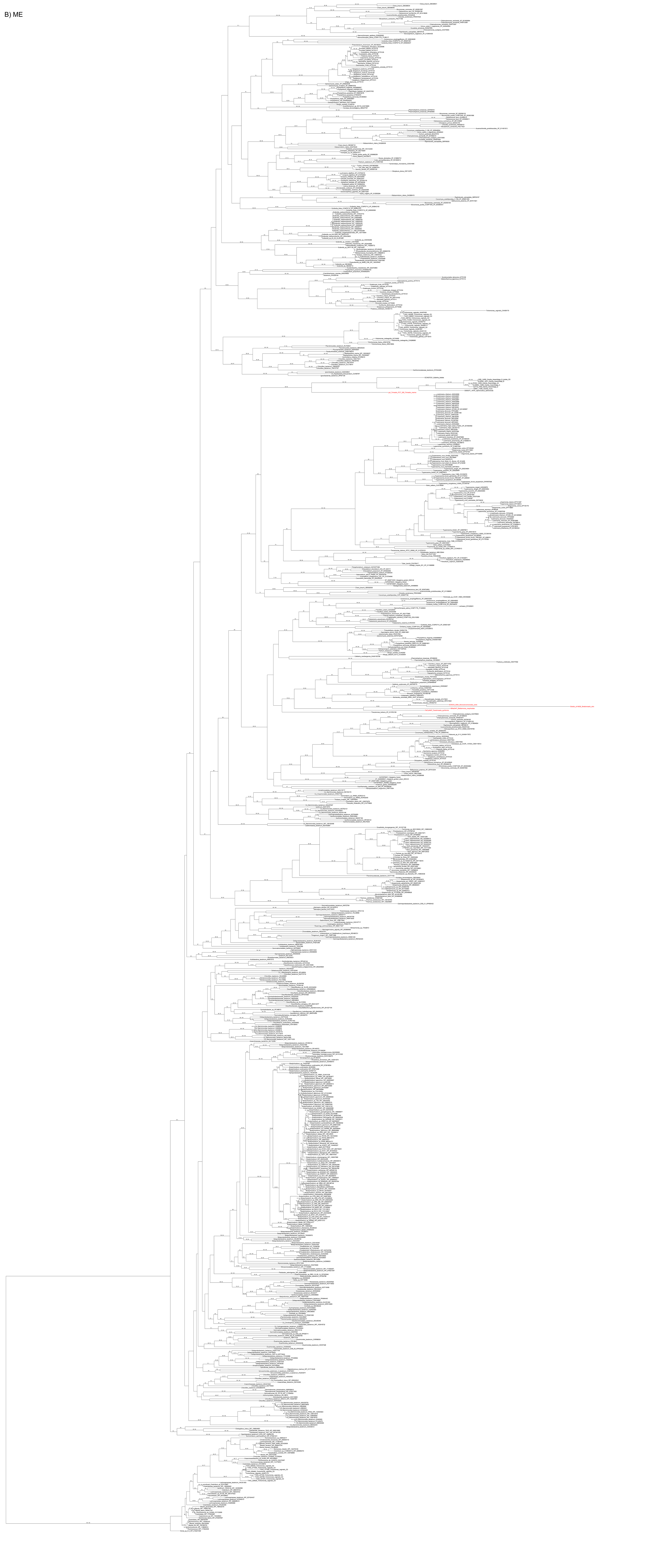

C) PFO

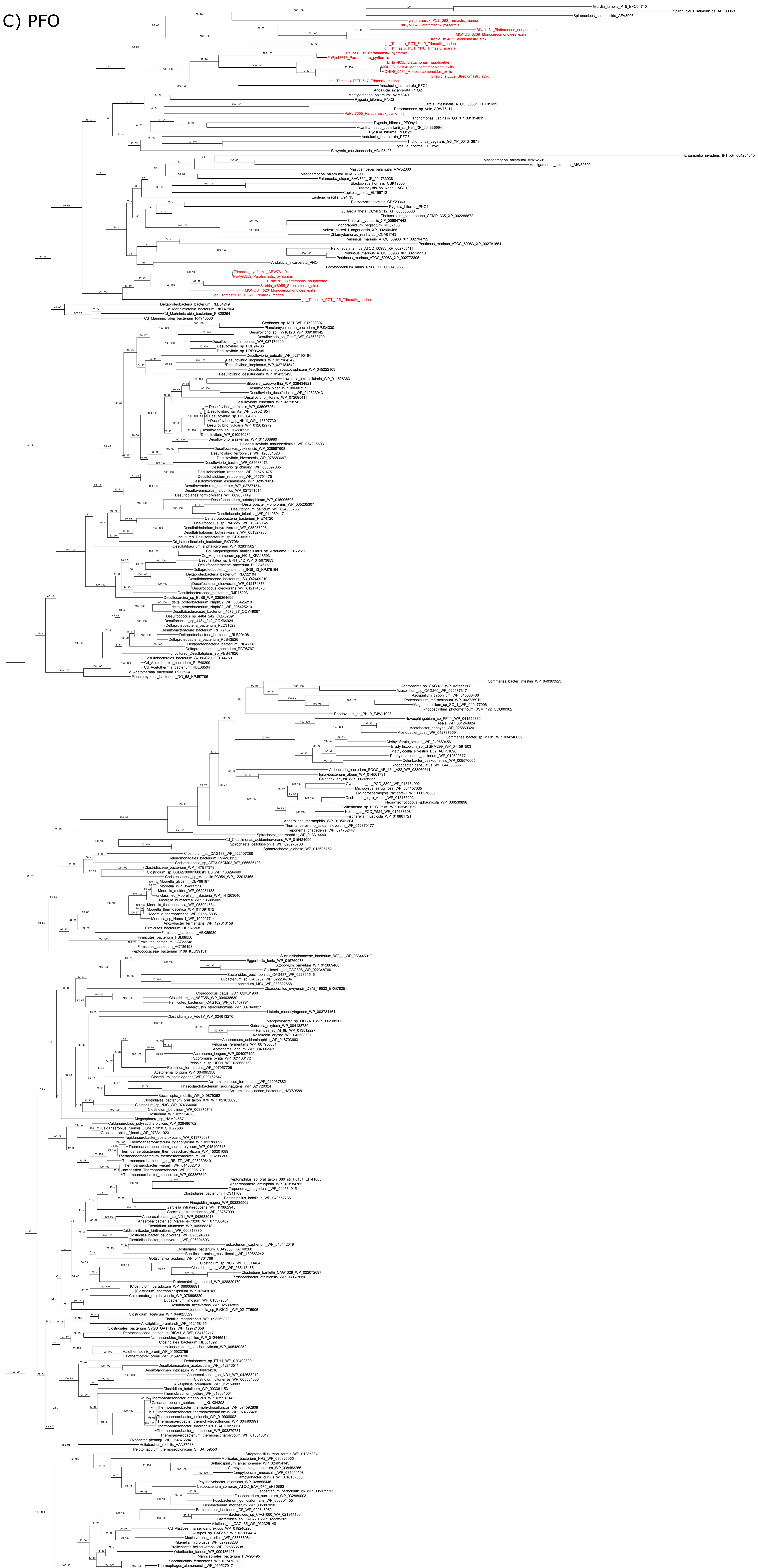

D) HydA

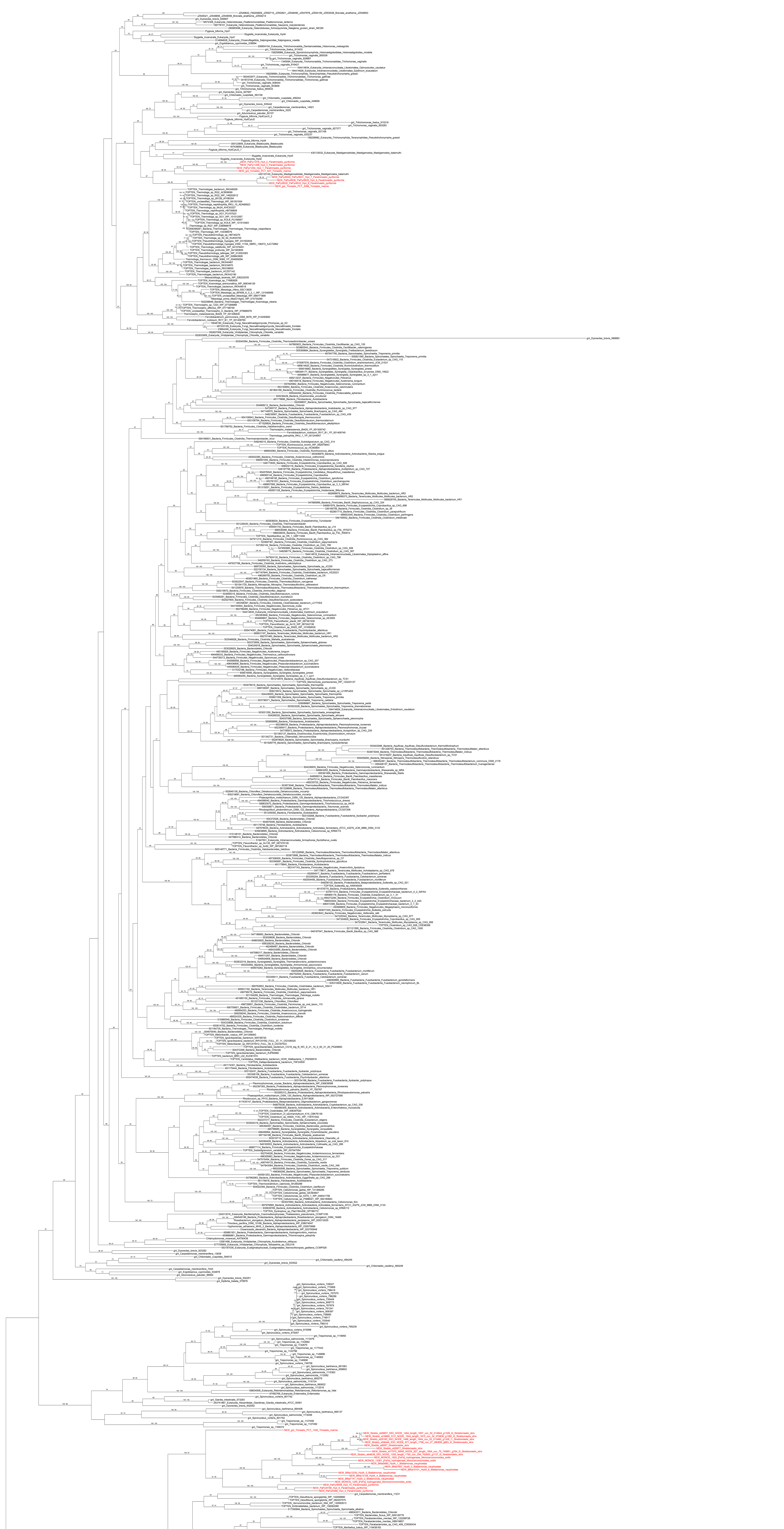

E) ACS

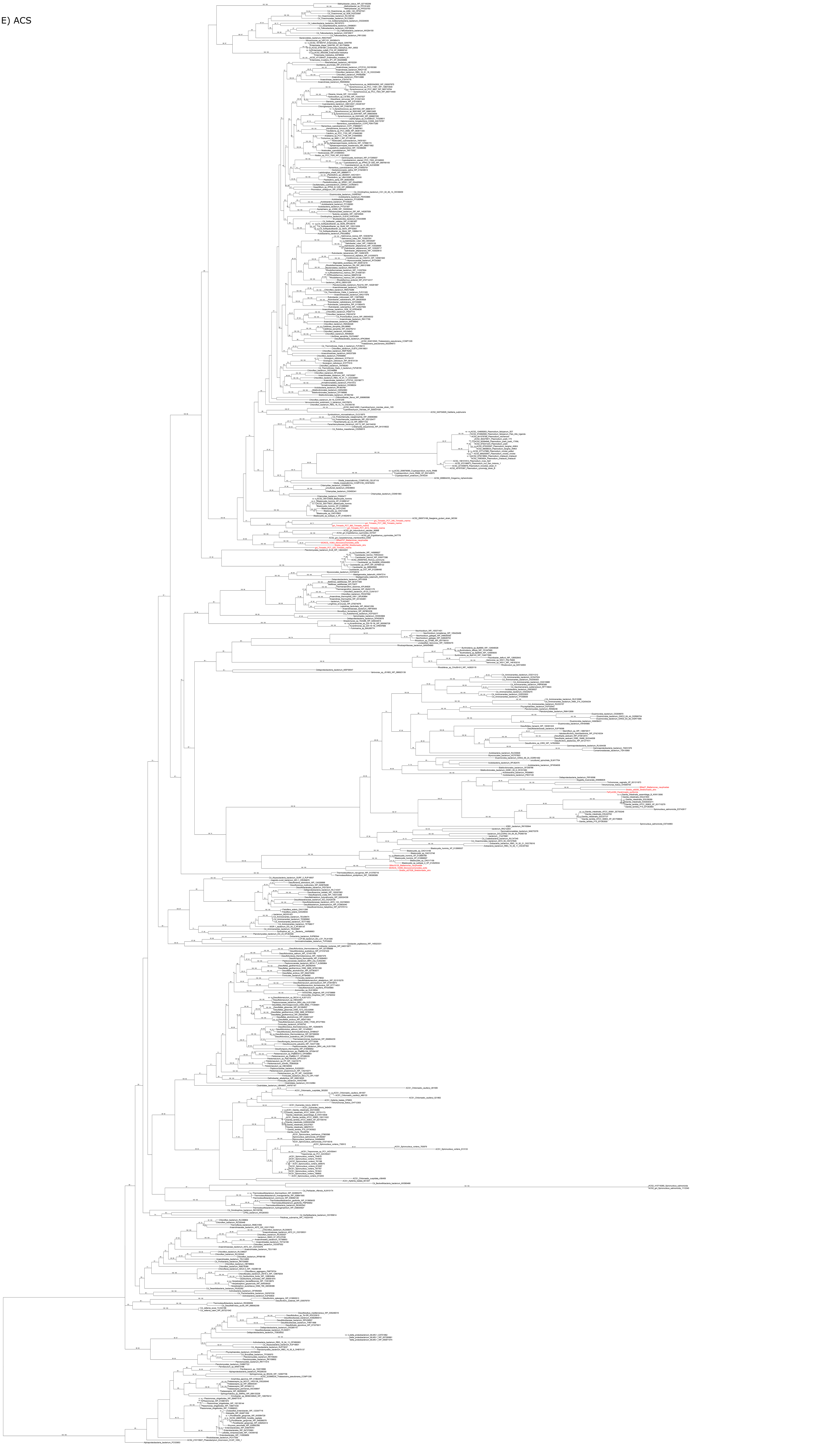

F) GCS L

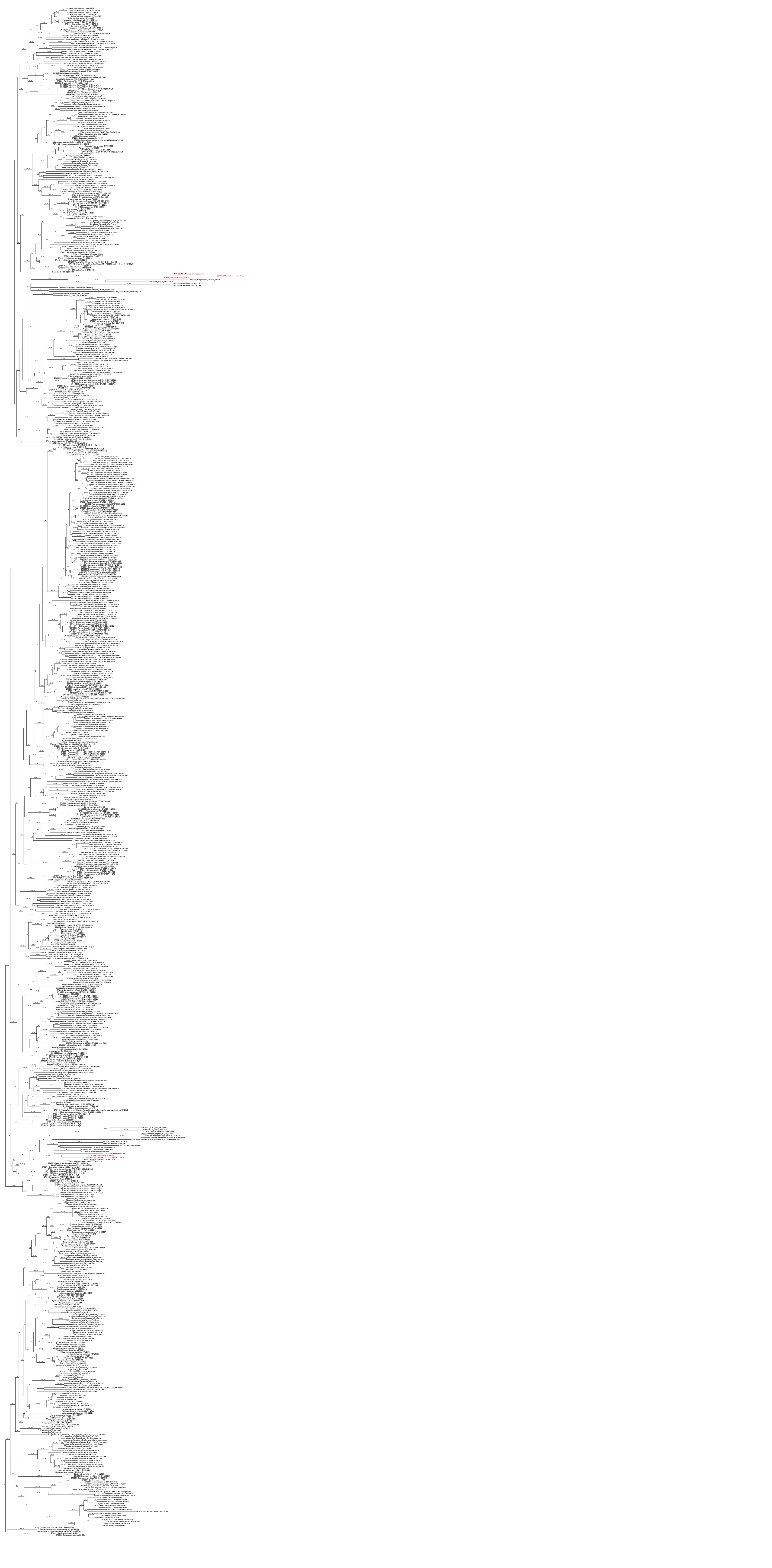

G) AAAP

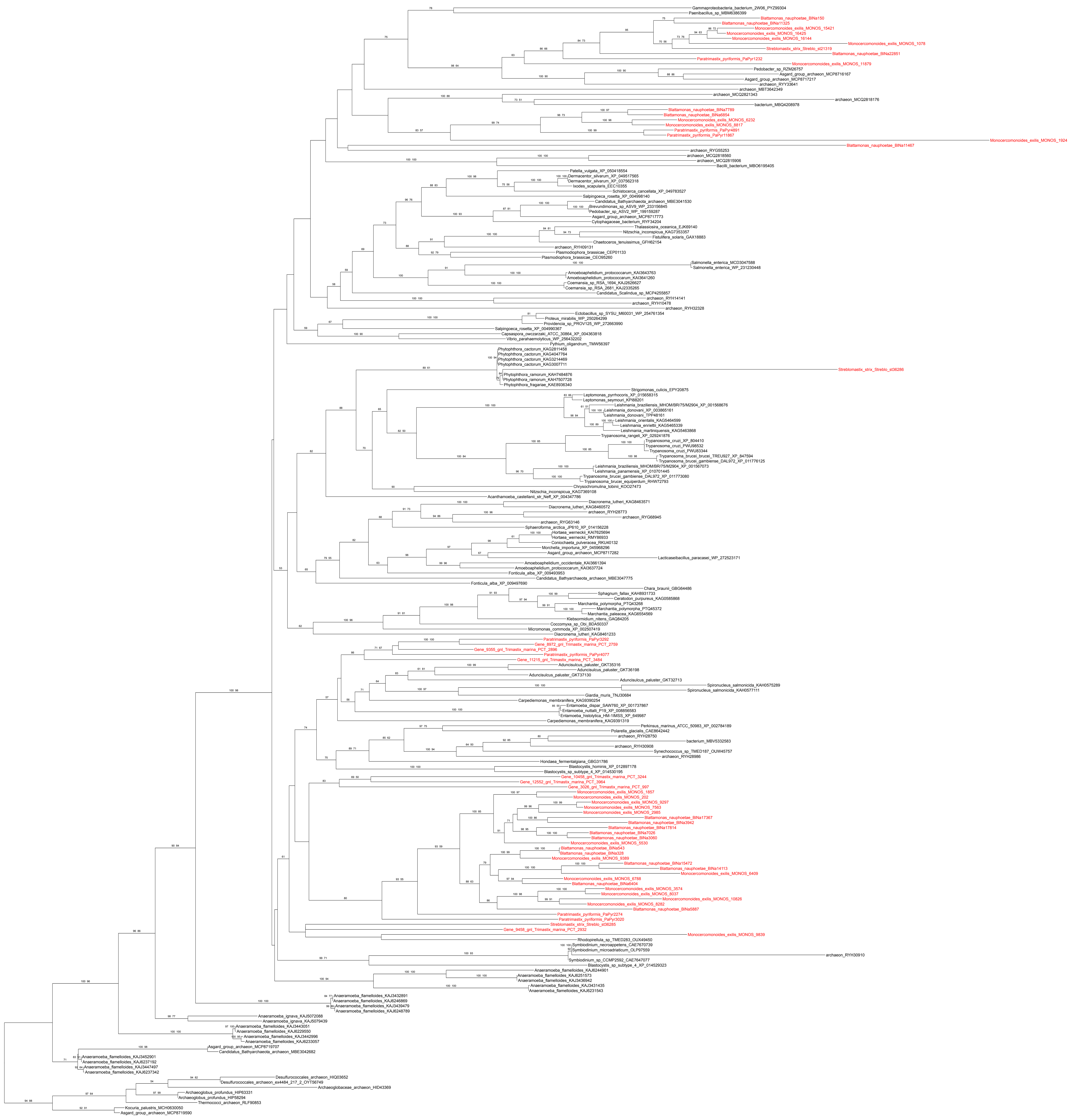

H) CTL

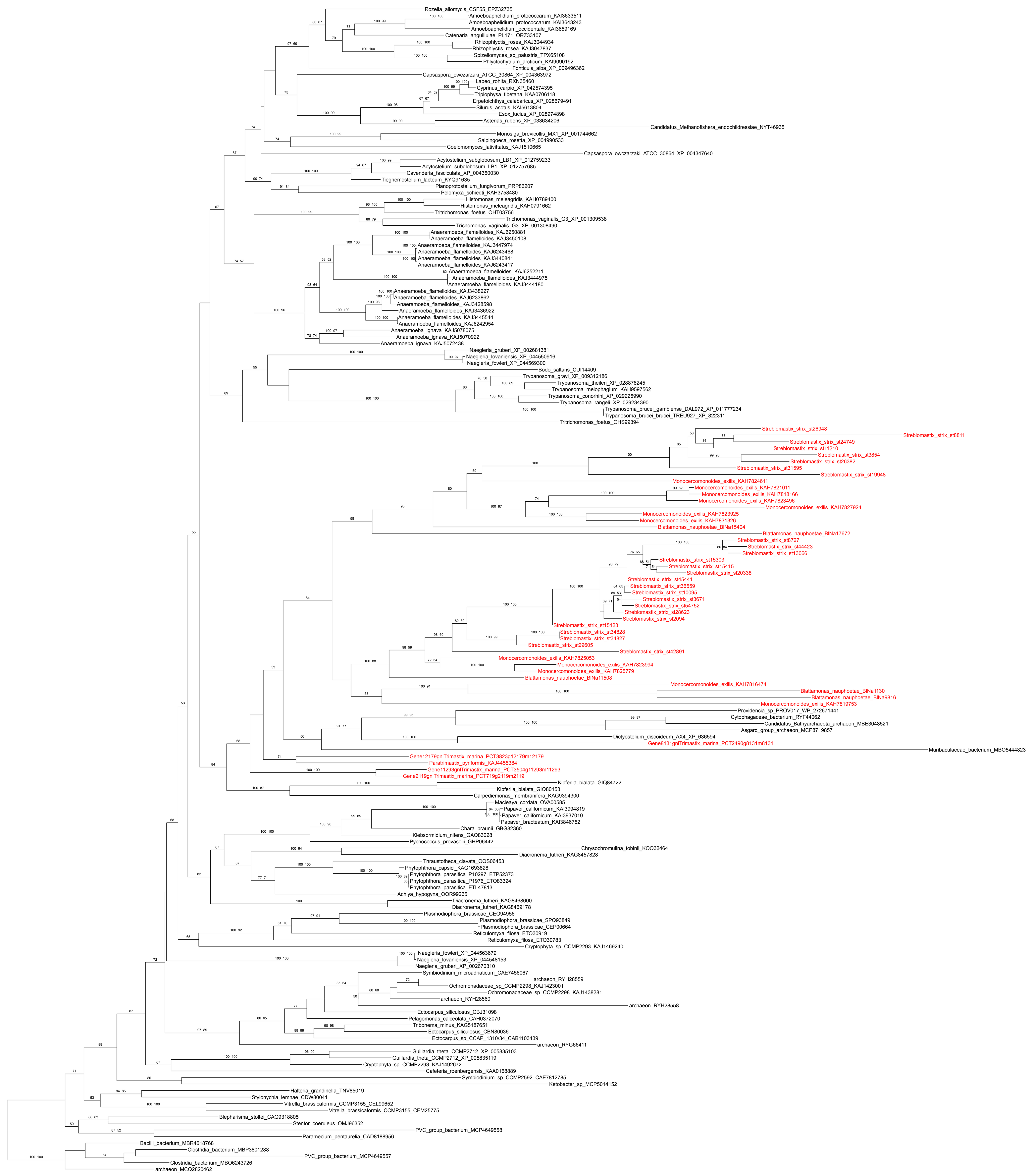

I) ENT

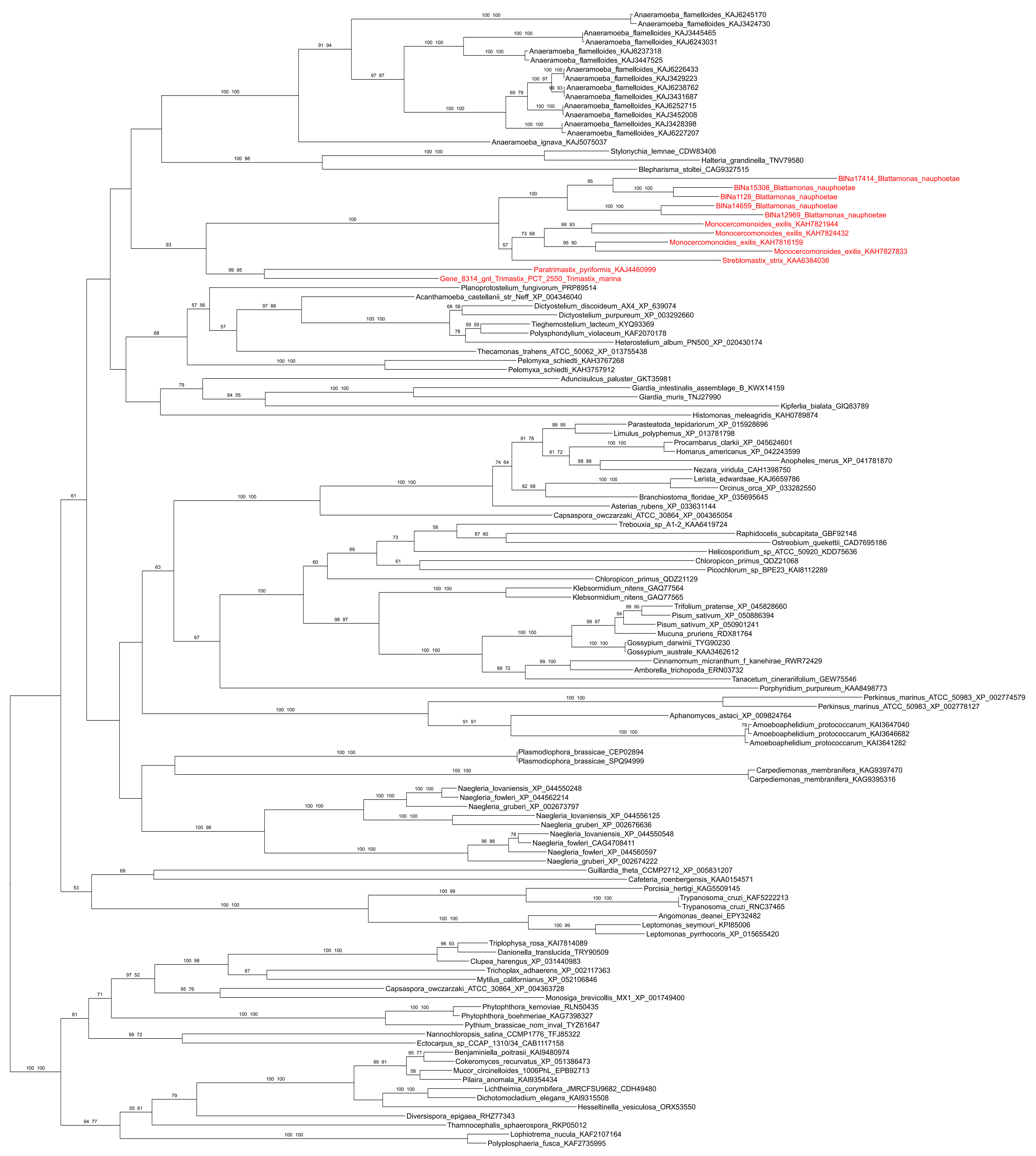

J) pATPase

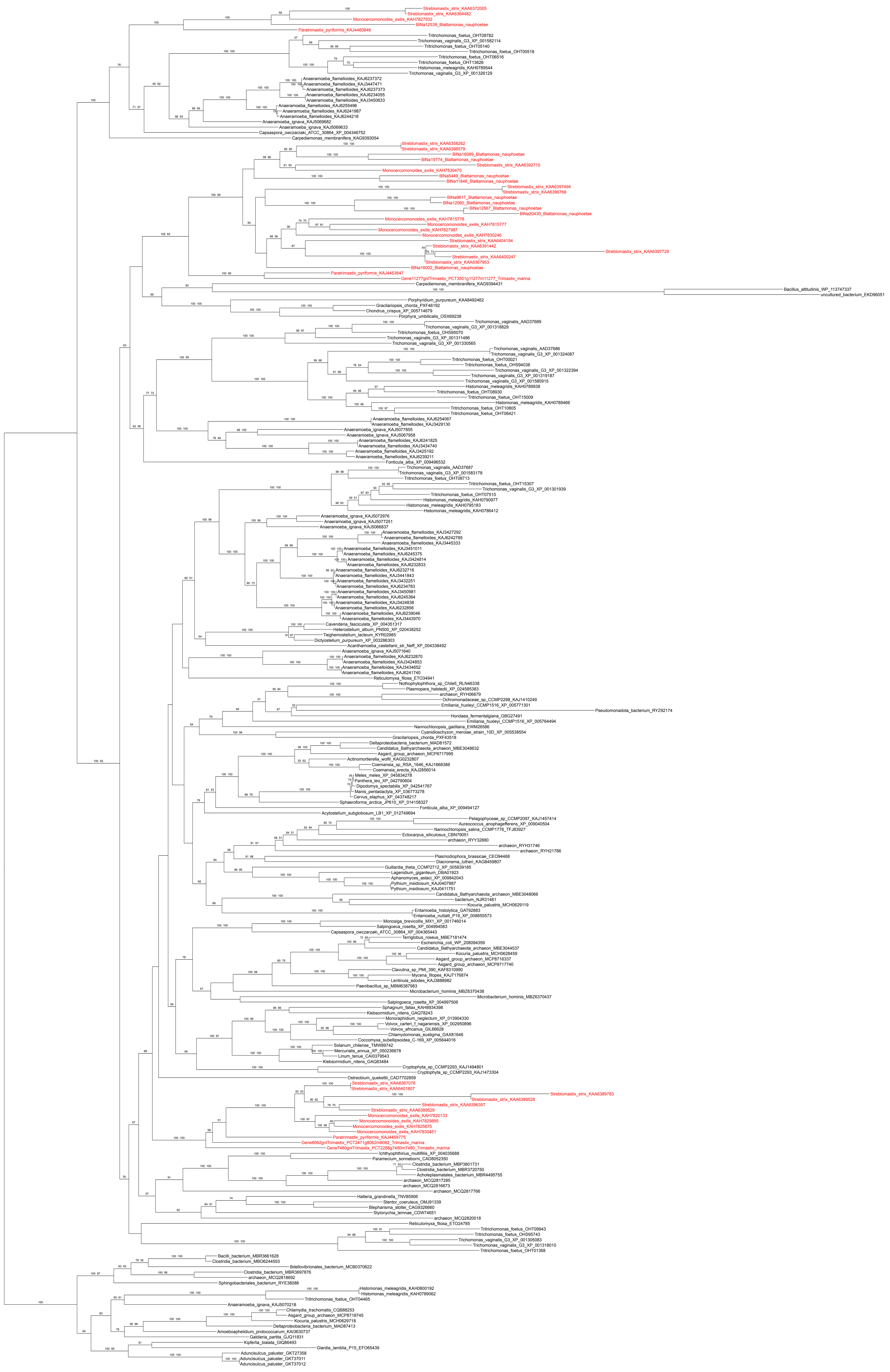

K) SP

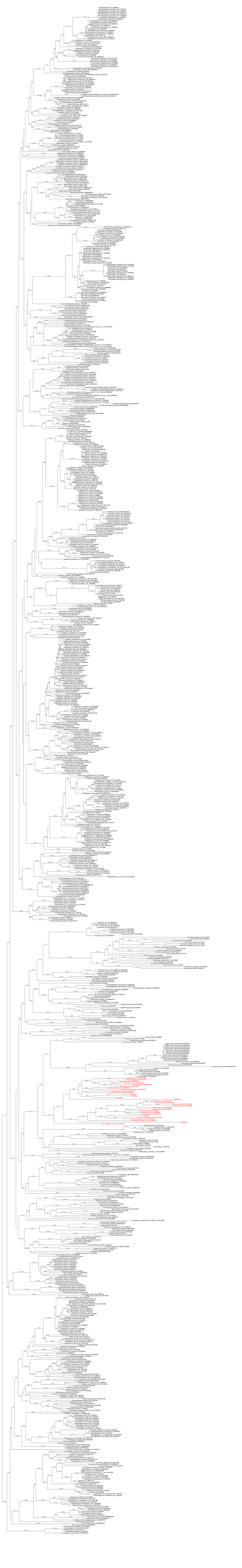

Supplement: S4 File — (PDF) [file pgen.1011050.s008.pdf]
