# Supplementary material for: Genomics of Preaxostyla Flagellates Illuminates the Path Towards the Loss of Mitochondria
Source: PLoS Genet. 2023 Dec 7;19(12):e1011050. doi: 10.1371/journal.pgen.1011050 (PMC10703272; doi:10.1371/journal.pgen.1011050)
Supplement: Attachment — Submitted filename: 132671_1_rebuttal_2693651_rvjdhp.pdf [file pgen.1011050.s014.pdf]

Full Revision

Review COMMONS

Manuscript number: RC-2022-01793
Corresponding author(s): Vladimír, Hampl

1. General Statements

Dear Editor and reviewers,

Thank you very much for the thorough assessment of our manuscript. We have carefully considered the comments and reflected most of them in the new version. We recognized the need to shorten and clarify the manuscript. Therefore, we have omitted particularly the less important passages concerning metabolism and the loss of genes encoding mitochondrial proteins, which cut the text by six pages in the current layout. We have also removed the text relating this model to eukaryogenesis. Finally, we have slightly changed the structure and linked the different sections to improve the flow of the story and to emphasize the key messages, which are the absence of mitochondria in a large proportion of oxymonads and the impact of this loss, loss of Golgi stacking and transformation to endobiotic lifestyle on selected gene inventories. We hope the manuscript is now clear and more concise and will be of interest to a broad readership interested in the evolution of eukaryotes, mitochondria and protists.

2. Point-by-point description of the revisions

The reviewer comments are shown in blue our answers in black.

Reviewer #1 (Evidence, reproducibility and clarity):

This is a very interesting paper that investigates through detailed comparative genomics the tempo and mode of the evolution of microbial eukaryotes/protists members of the Metamonada with a focus on Preaxostyla, currently the only known lineage among eukaryotes to have species that have lost, by all accounts, the mitochondria organelle all together. Notably, it includes a free-living representative of the lineage allowing potential interesting comparison between lifestyles among the Preaxostyla. This is a generally nicely crafted manuscript that presents well supported conclusions based on good quality genome sequence assemblies and careful annotations. The manuscript presents in particular (i) additional evidence for the common role of LGT from various bacterial sources into eukaryotic lineages and (ii) more details

on the transition from a free-living lifestyle to an endobiotic one and (iii) the related evolution of MROs and associated metabolism.

Thank you very much for the positive assessment.

I have some comments to improve a few details:

In the introduction, lines 42-43, the last sentence should be more conservative by replacing "whole Oxymonadida" with "...all known/investigated Oxymonadida".

The sentence has been changed to: "*Our results provide insights into the metabolic and endomembrane evolution, but most strikingly the data confirm the complete loss of mitochondria and every protein that has ever participated in the mitochondrion function for all three oxymonad species (M. exilis, B. nauphoetae, and Streblomastix strix) extending the amitochondriate status to all investigated Oxymonadida.*"

Similarly on line 62, the sentence could state "... contain 140 described...".

The sentence has been changed to: "*Oxymonadida contain approximately 140 described species of morphologically divergent and diverse flagellates exclusively inhabiting digestive tracts of metazoans, of which none has been shown to possess a mitochondrion by cytological investigations (Hampl 2017).*"

When discussing the estimated completeness of the genome are discussed (lines 117-120) and contrasted with the values for Trypanosoma brucei and other genomes, the author should explicitly state that these genomes are considered complete, which seems is what they imply, is that the case? If so, please provide more details to support this idea.

We have elaborated on this part also in reaction to comments of other reviewers. The text now reads: "*It should be noted that, despite their wide usage, BUSCO values are not expected to reach 100% in lineages distant from model eukaryotes simply due to the true absence (or high sequence divergence) of some of the assessed marker genes. For example, various Euglenozoa representatives with highly complete genome sequences, including Trypanosoma brucei, have BUSCO completeness estimates in the range of 71-88% (Butenko et al. 2020), and representatives of Metamonada fall within the range of 60-91% (Salas-Leiva et al. 2021). Specifically in the case of oxymonad M. exilis, the improvement of the genome assembly using long-read resequencing from 2092 scaffolds to 101 contigs led to only a marginal increase of BUSCO value from 75.3 to 77.5 (Treitli et al. 2021).* "

Also please see the detailed table prepared in response to reviewers 2 and 3 summarizing the presence/absence of genes from BUSCO set in the selected representatives of Metamonada and *Trypanosoma brucei*. The table is commented in the answer to Reviewer 3 comment (page

18)

The supplementary file named "132671_0_supp_2540708_rmsn23" is listed as a Table SX? (note: I found it rather difficult to establish exactly what file corresponds to what document referred in the main text)

We apologize for this mistake. We have checked and corrected references to tables, figures and supplementary material throughout the manuscript and hope it now does not contain any errors.

Lines 243-245, where 46 LGTs are discussed, it is relevant that the authors investigate their functional annotations. Indeed, it is suggested that these could have adaptive values, hence investigating their functional annotation will allow the authors to comment on this possibility in more details and precision. When discussing LGTs it would also be very useful to cite relevant reviews on the topic - covering their origins, functional relevance when known, distribution among eukaryotes. This is done when discussing the evolution and characteristics of MROs but not when discussing LGTs, with several reviews cited and integrated in the discussion of the data and their interpretation.

Available annotations of all putative LGT genes are provided in Supplementary_file_3 and also in the Supplementary_file_6 if the gene belongs to a manually annotated cellular system. Although we agree with the reviewer that the discussion of 46 species-specific LGTs might be interesting, for the sake of conciseness and brevity of the manuscript, we have decided not to expand the discussion further. However, note that we discuss selected cases of *P. pyriformis*-specific LGTs in the part "*P. pyriformis possesses unexpected metabolic capacities*" which follows right after the lines reviewer is referring to.

The sentence, lines 263-265, where the distribution of some LGTs are discussed, needs to be made more precise. When using the work "close" the authors presumably refer to shared/similar habitat,s or else? Entamoeba is not a close relative to the other listed taxa.

The "close relatives" mentioned in the text were meant as close relatives of all *p*-cresol-synthesizing taxa discussed in the paragraph, including *Mastigamoeba*, i.e. a specific relative of *Entamoeba*. We have modified the text such as to make the intended meaning easier to follow.

Lines 346-348, that sentence needs to end with a citation (e.g. Carlton et al. 2007).

The citation proposed by the reviewer has been added. The sentence was changed to: " *The most gene-rich group of membrane transporters identified in Preaxostyla is the ATP-binding cassette (ABC) superfamily represented by MRP and pATPase families, just like in T. vaginalis (Carlton et al. 2007).* "

In the paragraph (line 580-585) discussing ATP transporters, note that Major et al. (2017) did not describes NTTs but distantly related members of MSF transporter, shared across a broader

range of organisms then the NTTs. Did the authors check if the genome of interest encoded homologues of these transporters too?

The citation has been removed; we admit that it was not the most appropriate one in the given context. Concerning the NTT-like transporters, encouraged by the reviewer we searched for them in the Preaxostyla genome and transcriptome assemblies and found no candidates. This is not explicitly stated in the revised manuscript. The paragraph now reads: “*MROs export or import ATP and other metabolites typically using transporters from the mitochondrial carrier family (MCF) or sporadically by the bacterial-type (NTT-like) nucleotide transporters (Tsaousis et al. 2008). We did not identify any homolog of genes encoding proteins from these two families in any of the three oxymonads investigated. In contrast, MCF carriers, but not NTT-like nucleotide transporters, were recovered in the number of four for each P. pyriformis and T. marina (Supplementary file 6).*“

Line 920-921, I don't understand how the number 30 relates to "guarantee" inferring the directionality of LGTs events. This will be very much dataset dependent, 100 sequences might still not allow to infer directionality of LGT events. The authors probably meant to "increase the possibility to infer directionality".

We agree the original wording has not been particularly fortunate, so the sentence has changed to: "*Files with 30 sequences or fewer were discarded, as the chance directionality of the transfer can be determined with any confidence is low when the gene family is represented by a small number of representatives.*"

Reviewer #2 (Evidence, reproducibility and clarity):

Using draft genome sequencing of the free-living Paratrimastix pyriformis and the sister lineage oxymonad Blattamonas nauphoetae, Novack et al. infer the metabolic potential of the two protists using comparative genomics. The authors conclude that the common oxymonad ancestor lost the mitochondrion/mitosome and discuss general strategies for adapting to commensal/symbiotic life-style employed by this taxon. Some elaborations on pathways go on for several paragraphs and feel unnecessarily stretched, which made those sections of the paper rather difficult to digest.

Having seen reflections on the manuscript by three reviewers we carefully reconsidered its content and attempted to make it shorter and more compact by removing some of the less substantial material. Namely, we have dispensed completely with the original last section of Results and Discussion (“No evidence for subcellular retargeting of ancestral mitochondrial proteins in oxymonads”) and made various cuts throughout other sections. We hope that the

revised version makes a substantially better job of delivering the key messages of our study to the readers compared to the original submission.

This might be also be because the work, and all conclusions drawn, depend entirely on incomplete (ca. 70-80%) genome data and simple similarity searches, and e.g. no kind of biochemistry or imaging is presented to underpin the manuscripts discussion.

This is a very crude and superficial assessment of our data. We have actually good reasons to believe that the genome assemblies are close to complete. Please see the discussion on this topic below and an answer to a particular comment from reviewer 3 (page 18).

This is noteworthy in light of other protist genome reports published in the last few years that differ in this respect, including previous work by this group. And for sequencing-only data, this paper - https://doi.org/10.1016/j.dib.2023.108990 - might offer an example of where we are at in 2023.

Frankly, we do not think it is fair or relevant to compare our study to the paper pointed to by the reviewer, as that paper reports on a metagenomic study that delivers a set of metagenomically assembled genomes (MAGs) of varying quality retrieved from environmental DNA samples without providing any in-depth analysis of the gene content. Our study is very different in its scope and aims, and we are not certain what lesson we should take from this reviewer’s point. We have good reasons to believe that the datasets are close to complete. Please see the discussion on this topic below and answer to comment of reviewer 3 (page 18).

With respect to previous work of the group (Karnkowska et al. 2016 and 2019), this submission is very similar (analysis pattern, even some figures and more or less the conclusion), i.e. to say, the overall progress for the broader audience is rather incremental. Then there are also some incidents, where the data presented conflicts with the author‘s own interpretation.

It was our intention to use the previous analytical experiences and approaches, which at the same time makes the new results comparable with those published before. Although the format is intentionally similar, this work is a substantial step forward because only with our present study the amitochondrial status of the large part of Oxymonadida group can be considered solidly established. This in turn allows us to estimate the timing of the loss of mitochondrion (more than 100 MYA) demonstrating that the absence of mitochondrion in this group is not an episodic transient state but a long-established status. We do not understand what exactly the

reviewer had in mind when pointing to “incidents, where the data presented conflicts with the author‘s own interpretation” – we are not aware of such cases.

The text (including spelling and grammar) needs some attention and the choice of words is sometimes awkward. The overuse of quotation marks ("classical", "simple", "fused", "hits", "candidate") is confusing (e.g. was the BLAST result a hit or a "hit").

The whole text has been carefully checked and the language corrected whenever necessary by a one of the co-authors, who is a native English speaker. The use of quotation marks has been restricted as per the reviewer’s recommendation.

In its current formn the manuscript is, unfortunately, very difficult to review. This reviewer had to make considerable efforts to go through this very large manuscript, mainly because of issues affecting to the presentation and the lack of clarity and conciseness of the text. It would be greatly appreciated if the authors would make more efforts upfront, before submission, to make their work more easily accessible both to readers and facilitate the task of the reviewers.

We admit that the story we are trying to tell is a complex one, consisting of multiple pieces whose integration into a coherent whole is a challenging task. As stated above, the reports provided by the reviewers provided us with an important stimulus, leading us to substantially modify the manuscript to make it more concise, less ambiguous when it comes to particular claims, and easier to read. We hope this intention has been fulfilled to a larger degree.

About a fifth of the two genome is missing according the authors prediction (table 1). Early on they explain the (estimated) incompleteness of the genomes to be a result from core genes being highly divergent. In light of this already suspected high divergence, using (the simplest NCBI) sequence similarity approach to call out the absence of proteins (for any given lineage) may need lineage-specific optimization. The use of more structural motif-guided approaches such as hidden Markov models could help, but it is not clear whether it was used throughout or only for the search for (missing) mitochondrial import and maturation machinery. The authors state that the low completeness numbers are common among protists, which, if true, raises several questions: how useful are then such tools/estimates to begin with and does this then not render some core conclusions problematic? The reader is just left with this speculation in the absence of any plausible explanation except for some references on other species for which, again, no context is provided. Do they have similar issues such as GC-content, same core genes missing, phylogenetic relevance?, etc.. No info is provided, the reader is expected to simply accept this as a fact and then also accept the fact that despite this flaw, all conclusions of the paper that rests on the presence/absence of genes are fine. This is all odd and further skews the interpretations and the comparative nature of the paper.

The question of the completeness of the data sets was raised also by reviewer 3 and we would like to provide an explanation at this point. First, it should be stated that there is no ideal and objective way how to measure the completeness of the eukaryotic genomic assembly. In the

manuscript, we have used the best established method, adopted by the community at large, which is based on the search for a set of „core eukaryotic genes“ using a standardized pipeline BUSCO or previously popular CEGMA. The pipeline uses its own tools to identify the homologues of genes/proteins which ensures standardization of the procedure. This answers the question of reviewer 2, why we have not used more sensitive tools for these searches. We did not use them, because we followed the procedure that is the gold standard for such assessments, for comparability with other genomes and to make this as clear to the reader as possible. Although the result of the pipeline is usually interpreted as the completeness of the assembly, this is a simplification. Strictly speaking, the result is a percentage of the genes from the set of 303 core eukaryotic genes (in our case) which were detected in the assembly by the pipeline. Even in complete assemblies, the value is usually below 100% because some of the genes are not present in the organism and some diverged beyond recognition. We do not see any other way how to deal with this drawback than to compare with related complete genome assemblies acting as standards. This we have done in Supplementary file 11, where we list the presence/absence of each gene for Preaxostyla species and three highly complete assemblies of *Trypanosoma brucei, Giardia intestinalis* and *Trichomonas vaginalis*. *T. brucei* and *G. intestinalis* are assembled into chromosomes. As you can see, in these three „standards“ 63, 148 and 77 genes from the core were not detected resulting in BUSCO completeness values of 79%, 51% and 75%, respectively. 18 of the non-detected genes function in mitochondria (shown in red), which are highly reduced in some of these species, so the absence of the respective genes is therefore expected. Simply not considering these genes would increase the “completeness measure” for oxymonads by 6%. The values for our standards are not higher than the values for Preaxostyla (69-82%). In summary, the BUSCO incompleteness measure is far from ideal, particularly in these obscure groups of eukaryotes. The values received for Preaxostyla give no reason for concern about their incompleteness. See also our answer to reviewer 3 (page 18).

At the same time, we admit that the BUSCO values do not confirm the high completeness of our assemblies. So, why do we think they are highly complete? One reason is that we do not see suspicious gaps in any of the many pathways which we annotated but the main reason is the high contiguity of the assemblies. Thanks to Nanopore long read sequencing, the assembly of *P. pyriformis* and *B. nauphoetae* compose of 633 and 879 scaffolds, suggesting that there are “only” hundreds of gaps. Although this may still sound too much, it is a relatively good achievement for genomes of this size and the experience shows that a further decrease in the number of scaffolds would allow the detection of additional genes but not in huge numbers. As we have shown for *M. exilis* (Treitli et al. 2021, doi:10.1099/mgen.0.000745) the decrease from 2 092 scaffolds to 101 contigs, i.e., filling almost 2 000 gaps, allowed the prediction of additional 1 829 complete gene models, of which 1 714 were already present in the previous assembly but only partially and just 115 were completely new. None of these newly predicted genes was

functionally related to the mitochondrion. Thus, we infer the chance that all mitochondrion-related genes are hidden in the gaps of assemblies is very low.

We have provided these arguments in a condensed form in the text following the description of genome assemblies: "*It should be noted that, despite their wide usage, BUSCO values are not expected to reach 100% in lineages distant from model eukaryotes simply due to the true absence (or high sequence divergence) of some of the assessed marker genes. For example, various Euglenozoa representatives with highly complete genome sequences, including Trypanosoma brucei, have BUSCO completeness estimates in the range of 71-88% (Butenko et al. 2020), and representatives of Metamonada fall within the range of 60-91% (Salas-Leiva et al. 2021). Specifically in the case of oxymonad M. exilis, the improvement of the genome assembly using long-read resequencing from 2092 scaffolds to 101 contigs led to only a marginal increase of BUSCO value from 75.3 to 77.5 (Treitli et al. 2021).*"

As a side note, this will also influence the number of proteins absent in other lineages and as such has consequences on LGT calls versus de novo invention. For the cases with LGT as an explanation, it would help to briefly discuss the candidate donors and some details of the proteins in the eco-physiological context (e.g. lines 263-268 suggest that HPAD may have been acquired by EGT which was facilitated by a shared anaerobic habitat and also comment on adaptive values for acquiring this gene). Exchanging metabolic genes via LGT (Line 163) blurs the differences between roles and extent of LGT in prokaryote vs eukaryote, and therefore is exciting and could use support/arguments other than phylogenies. I guess the number of reported LGTs among protists (whatever the source) over the last decade has by now deflated the novelty of the issue in more general; a report of the numbers is expected but they alone won't get you far anymore in the absence of a good story (such as e.g. work on plant cell wall degrading enzymes in beetles).

We agree with the reviewer that the cases of LGT involving Preaxostyla would deserve more discussion in the manuscript. On the other hand, we also agree that none of them provides such a "cool" story that would deserve a special chapter or even a separate paper. Therefore, we have decided, also with regard to keeping the text in a reasonable dimension, not to expand the discussion of LGTs with the exception of HgcAB, where some new information has been included and the phylogeny of the genes updated. Please note that we had discussed in the original manuscript the donor lineages and ecological/biochemical context in the cases of GCS-L2, HPAD, UbiE, and NAD+ synthesis and this material has been kept also in the revised version.

It would help to clarify which parts of the mitochondrial ancestor were reduced during the process of reductive evolution at what time in their hypothesized trajectory. For instance, loosing enzymes of anaerobic metabolism conflicts with the argued case of an aerobic (as opposed to facultative anaerobic) mitochondrial ancestor followed by gains of anaerobic metabolism in the rest of the eukaryotes via LGT, and some papers the authors themselves cite (e.g. the series by

Stairs et al.). There is no coherent picture on LGT and anaerobic metabolism, although a reader is right to expect one.

These are very interesting questions, that would fill a separate article. In the manuscript, we focus on the Preaxostyla lineage only and there the trajectory seems relatively simple: replacement of the mitochondrial ISC by cytosolic SUF in the common ancestor of Preaxostyla, loss of methionine cycle and in in consequence mitochondrial GCS and the mitochondrion itself. We have modified the first conclusion paragraph in this sense and it now reads the following:

"The switch to the SUF pathway in these species has apparently not affected the number of Fe-S-containing proteins but led to a decrease in the usage of 2Fe-2S clusters. The loss of MRO impacted particularly the pathways of amino acid metabolism and might relate also to the loss of large hydrogenases in oxymonads. "

It is not clear to us how to understand the reviewer's remark concerning the conflict between loss of enzymes of anaerobic metabolism and the (presumed) aerobic nature of the mitochondrial ancestor. Provided that we read the reviewer's rationale correctly, is it really so implausible that the anaerobic metabolism gained laterally by a particular lineage was then secondarily lost in specific descendant lineages? As a clear example demonstrating the feasibility of such an evolutionary pattern consider the evolution of plastids. There is no doubt these organelles move across eukaryotes by secondary or higher-order endosymbiosis or kletoplastidy, establishing themselves in lineages where there was no plastid before. Secondary simplification of such plastids, e.g. by the loss of photosynthesis, in its extreme form culminating in the complete loss of the organelle, has been robustly documented from several lineages, such as Myzozoa (e.g., https://pubmed.ncbi.nlm.nih.gov/36610734/). Hence, we see absolutely no reason to rule out the possibility that the ancestral mitochondrion was obligately aerobic and enzymes of anaerobic metabolism spread secondarily by eukaryote-to-eukaryote LGT, with their secondary loss in particular lineages. We really do not see any conflict here and we do not agree with the interpretation provided by the reviewer. That said, we admit that the discussion on the earliest stages of mitochondrial evolution is not an essential ingredient of the story we try to tell in our manuscript, so to avoid any unnecessary misunderstanding we have removed the original last sentence of Conclusions ("Thorough searches revealed …") from the revised manuscript.

In light of their data the authors also discuss the importance of the mitochondrion with respect to the origin of eukaryotes:

First, the mitochondrion brought thousands of genes into the marriage with an archaeon, surely hundreds of which provided the material to invent novel gene families through fusions and exon shuffling and some of which likely went back and forth over the >billion years of evolution with respect to localizations. The authors look at a minor subset of proteins (pretty much only those of protein import, Fig. 6) to conclude, in the abstract no less: „most strikingly the data confirm the complete loss of mitochondria and every protein that has ever participated in the

mitochondrion function for all three oxymonad species." I do not question the lack of a mitochondrion here, but this abstract sentence is theatrical in nature, nothing that data on an extant species could ever proof in the absence of a time machine, and is evolutionary pretty much impossible. A puzzling sentence to read in an abstract and endosymbiont-associated evolution.

We feel that the reviewer is putting too much emphasis on an aspect of our original manuscript that is rather peripheral to its major message. Indeed, the manuscript is not, and has never been thought to be, primarily about eukaryogenesis and the exact role the mitochondrion played in it. We are, therefore, somewhat reluctant to react in full to the very long and complex argument the reviewer has raised in his/her report, so we keep our reaction at the necessary minimum. Concerning the criticized sentence in the original version of the abstract, it alluded to a section of the manuscript ("No evidence for subcellular retargeting of ancestral mitochondrial proteins in oxymonads") that we have removed from the revised version, and hence we have modified also the abstract accordingly by removing the sentence. We still think our original arguments were valid, but apparently, much more space and more detailed analyses are required to deliver a truly convincing case, for which there is no space in the manuscript.

Second, using oxymonads as an example that a lineage can present eukaryotic complexity in the absence of mitochondria and conflating it with eukaryogenesis is a logical fallacy. This issue already affected the 2019 study by Hampl et al.. We have known that a eukaryote can survive without an ATP-synthesizing electron transport chain ever since Giardia and other similar examples and the loss of Fe-S biosynthesis and the last bit of mitosome (secondary loss) doesn't make a difference how to think about eukaryogenesis. It confuses the need and cost to invent XYZ with the need and cost of maintenance. How can the authors write "... and undergo pronounced morphological evolution", when they evidently observe the opposite and show so in their Fig. 1? The authors only present evidence for reductive evolution of cellular complexity with the loss of a stacked Golgi. What morphological complexity did oxymonads evolve that is absent in other protists? A cytosolic metabolic pathway doesn't count in this respect, because it is neither morphological, nor was it invented but likely gained through LGT according to the authors. This is quite confusing to say the least. A recent paper (https://doi.org/10.7554/eLife.81033) that refers to Hampl et al. 2019 has picked this up already, and I quote: "Such parasites or commensals have engaged an evolutionary path characterized by energetic dependency. Their complexity might diminish over evolutionary timescale, should they not go extinct with their hosts first." Here the authors raise a red flag with respect to using only parasites and commensals that rely on other eukaryotes with canonical mitochondria as examples. If we now look at Fig. 1 of this submission, Novak et al. underpin this point perfectly, as the origin of oxymonads is apparently connected to the strict dependency on another eukaryote (or am I wrong?), and they support the prediction with respect to complexity reducing after the loss of mitochondria - mitosome gone, Golgi almost gone. What's next? This is a good time to remember that extant oxymonads are only a single picture frame in the movie that is

evolution, and their evolution might be a dead-end or result in a prokaryote-like state should they survive 100.000s to millions of years to come.

It seems that in this point the reviewer is particularly concerned with the following sentence that is part of the Introduction and which relates to the existence of amitochondrial eukaryotes we are studying: "*The existence of such an organism implies that mitochondria are not necessary for the thriving of complex eukaryotic organisms, which also has important bearings to our thinking about the origin of eukaryotes (Hampl et al. 2018).*" Even after re-reading the sentence we confess we stay with it and find it perfectly logical. Nevertheless, we decided to omit it from the text so as not to distract from the main topic of the study.

Next, when mentioning "… pronounced morphological evolution" we mean the evolution of four oxymonad families (Streblomastigidae, Oxymonadidae, Pyrsonymphidae and Saccinobaculidae) comprising almost a hundred described species with often giant and morphologically elaborated cells that evolved from a simple *Trimastix*-like ancestor (Hampl 2017, Handbook of Protists, 0.1007/978-3-319-32669-6_8-1). This is a fact that can hardly be dismissed. Also, given the current oxymonad phylogenies (Treitli et al. 2018, doi.org/10.1016/j.protis.2018.06.005) and the reported absence of a mitochondrion in *M. exilis*, *B. nauphoetae*, and *S. strix* we can infer that the mitochondrion was lost in the common ancestor of the three species at latest. This organism must have lived more than 100 MYA, as at that time oxymonads were clearly diversified into the families (Poinar 2009, 10.1186/1756-3305-2-12). So, these organisms indeed have lived without mitochondria for at least 100 MY. We think that these facts and our inferences based on them are solid enough to keep in the conclusion the following statement: "*This fact moves this unique loss to at least 100 MYA deep past, when oxymonads had been already diversified (Poinar 2009), and shows that a eukaryotic lineage without mitochondria can thrive for eons and undergo pronounced morphological evolution, as is apparent from the range of shapes and specialized cellular structures exhibited by extant oxymonads (Hampl 2017).*" Furthermore, as documented in Karnkowska et al. 2019 (https://pubmed.ncbi.nlm.nih.gov/31387118/), apart the loss of the mitochondrion oxymonads are surprisingly "normal" and complex eukaryotes, in fact much less reduced than, e.g., *Giardia*, Microsporidia, or even *S. cerevisiae* (in terms of the number of genes, introns, etc.). We strongly disagree with the claim that "Golgi is almost gone" in oxymonads, and our manuscript shows exactly the opposite. Viewing oxymonads as a lineage heading towards a prokaryote-like simplicity is dogmatic and ignores the known biology of these organisms.

Some more thoughts:
Line 47-52: Hydrogenosome or mitosome is a biological and established label as (m)any other and I find the use of the word "artificial" in this context strange. While the authors are correct to note that there is a (evolutionary) continuum in the reduction - obviously it is step by step - they exaggerate by referring to the existing labels as "artificial". You make Fe-S clusters but produce no ATP? Well, then you're a mitosome. It's a nomenclature that was defined decades ago and has proven correct and works. If the authors think they have a better scheme and definition, then please present one. Using the authors logic, terms such as amyloplast or the TxSS

nomenclature for bacterial secretions systems are just as artificial. As is, this comes across as grumble for no good reason.

We agree that the original wording sounded like unwarranted grumbling and we have changed the sentence in the following way: "However, exploration of a broader diversity of MRO-containing lineages makes it clear that MROs of various organisms form a functional continuum (Stairs et al. 2015; Klinger et al. 2016; Leger et al. 2017; Brännström et al. 2022)."

Line 158: A duplication-divergence may also explain this since sequence similarity-based searches will miss the ancestral homologues.

We do not disagree about this, in fact, the gene the reviewer's point is concerned with for sure is a result of duplication and divergence, as it belongs to a broader gene family (major facilitator superfamily, as stated in the manuscript) together with other distant homologs. Nevertheless, this is not in conflict with our conclusion that it "*may represent an innovation arising in the common ancestor of Metamonada*".

Lines 201-202: Presence of GCS-L in amitochondriate should be explained in light of this group once having a mitochondrion, which then makes ancestral derivation and differential loss (as invoked for Rsg1) also a likely explanation along with eukaryote-to-eukaryote LGT.

Yes, this most likely holds for the standard paralogue GCS-L1 (in *P. pyriformis* PAPYR_5544), which has the expected distribution and phylogenetic relationships and is absent in oxymonads. The discussion is, however, mainly about the rare, divergent and until now overlooked paralogue GCS-L2 (in *P. pyriformis* PAPYR_1328), which we found only in three distantly related eukaryote groups, Preaxostyla, Breviatea, and Archamoebae, which strongly suggests inter-eukaryotic LGT.

Lines 356-392: Describes plenty of genomic signal for Golgi bodies but simultaneously cites literature suggesting the absence of a morphologically an identifiable Golgi in oxymonads. An explicit prediction regarding what to observe in TEM for the mentioned species might be nice to stimulate further work.

We thank the reviewer for their suggestion and are glad that they are enthusiastic about this aspect of the manuscript. Unfortunately, the morphology of unstacked Golgi ranges from single cisternae (yeast, *Entamoeba*), vesicles (*Mastigamoeba*), and a "tubular membranous structure" in *Naegleria*. Therefore, no strong prediction is possible of what the oxymonad Golgi might look like under light or TEM. However, the data that we have provided should lead to molecular cell biological analyses aimed at identifying the organelle, giving target proteins to tag or against which to create antibodies as Golgi markers. An additional sentence to this effect has been

added to the manuscript, “They also set the stage for molecular cell biological investigations of Golgi morphological variation, once robust tools for tagging in this lineage are developed.”

Lines 414: The preceding paragraphs in this result section describes only the distribution, without mentioning origins - a sweeping one-line summary that proclaims different origin needs some context and support. Furthermore, the distribution of glycolytic enzymes might indeed be patchy, but to suggest it represents an 'evolutionary mosaic composed of enzymes of different origins' without discussing the alternative of a singular origin and different evolutionary paths (including a stringer divergence in one vs. another species) discredits existing literature and the authors own claim with respect to why BUSCO might fail in protists.

The part of the text about glycolysis the reviewer alluded to has been removed while shortening the manuscript.

Line 486: How uncommon are ADI and OTC in lineages sister to metamonada?

This is an interesting but difficult question. Firstly, we are uncertain what is the sister lineage to Metamonada. Discoba, maybe, but a recent unpublished rooting of the eukaryotic tree does not support it (https://pubmed.ncbi.nlm.nih.gov/37115919/). Generally, the individual genes of the pathway (ADI, OTC and CK) are quite common in eukaryotes, but the combination of all three is rare (Metamonada, the heterolobosean *Harpagon*, the green algae *Coccomyxa* and *Chlorella*, the amoebozoan *Mastigamoeba*, and the breviate *Pygsuia*), see figure 1 in Novak et al 2016, doi: 10.1186/s12862-016-0771-4.

Line 504: It might help an outside reader to include a few lines on consequences and importance of having 2Fe-S vs 4Fe-S clusters and set an expectation (if any) in Oxymonads.

We apologize for omitting this explanation. The 2Fe-2S proteins are more common in mitochondria where 2Fe-2S clusters are synthesized in the early pathway of FeS cluster assembly, while the cytosolic CIA pathways produce 4Fe-4S clusters (https://pubmed.ncbi.nlm.nih.gov/33007329/). The original expectation therefore is that species without mitochondria should not have 2Fe-2S cluster proteins. Obviously, the switch to the SUF pathway affects this expectation as we do not know, what type of cluster this pathway produces in oxymonads (https://www.biorxiv.org/content/10.1101/2023.03.30.534840v1). For the sake of brevity, we have included a short statement as the beginning of the sentence in question, which now reads as follows: “*As 2Fe-2S clusters are more frequent in mitochondrial proteins, the higher number of 2Fe-2S proteins in P. pyriformis compared to the oxymonads may reflect the*

presence of the MRO in this organism."

Any explanations on what unique selection pressures and gene acquisition mechanisms may be operating in P. pyriformis which might allow for the unique metabolic potential?

Every species exhibits a unique combination of traits that results from changing selection pressures imposed on historical contingency (including neutral evolutionary processes such as genetic drift). We lack real understanding of these factors for a majority of taxa including the familiar ones, so we should not expect to have a good answer to the reviewer's question. In fact, we do not know how unique is the particular combination of *P. pyriformis* traits discussed in our manuscript, as there has been no comprehensive comparative analysis that would include ecologically and evolutionarily comparable taxa. We note that *Paratrimastix* represents only a third free-living metamonad with a sequenced genome (together with *Kipferlia* and *Carpediemonas*), so more data and additional analyses are needed to be in a position when we may start hoping answers to questions like the one posed by the reviewer are in reach.

** Referees cross-commenting**
To R3: Hampl et al. 2019, to which Novak et al. refer, is about eukaryogensis and that is exactly the context in which this is discussed again and what Raval et al. 2022 had decided to touch upon. If the authors do not bring this up in light of the ability to evolve (novel) eukaryote complexity, then what else? Maybe they can elaborate, especially with respect to energetics to which they explicitly refer to in 2019 (and here). And with respect to text-book eukaryotic traits (and the evolution of new morphological ones), I do not see any new ones evolving in any oxymonad, but reduction as Novak et al. themselves picture it in this submission. Is a change in the number of flagella pronounced morphological evolution? Maybe for some, but I believe this needs to be seen in light of the context of how they discuss it. I see a reduction of eukaryotic complexity and not a gain. They have an elaborate section on the loss of Golgi characteristics (and a figure), but I fail to read something along the same lines with respect to the gain of new morphological traits. Again, novel LGT-based biochemistry does not equal the invention of a new morphology such as a new compartment. Oxymonads depend on mitochondria-bearing eukaryotes for their survival or don't they? This is the main point, and if evidence show that I am wrong, then I will be the first to adapt my view to the data presented.

While we do see the logic of the reviewer's point, a good reply would have to be too elaborate and certainly beyond the scope of the current manuscript. As the reviewers' reports led us to reconsider the structure of the manuscript and to make it more focused and concise, we decided to simplify the matter by removing the allusions to eukaryogenesis, realizing that it is perhaps more suitable for a different type of paper (opinion, review). The comment on the evolution of complex morphology has been answered previously (see above).

I have concerns with the presentation of a narrative that in my opinion is too one-sided and that has been has been publicly questioned in the community (in press, at meetings, personally). For the benefit of science and of the young authors on this study, this reviewer feels strongly that

these issues should be taken very seriously and discussed openly in a more balanced way. . We only truly move forward on such complex topics, if we allow an open and transparent discussion.

We agree that opinions on specific details of eukaryogenesis are divided in the community and that the topic requires a nuanced discussion for which there is perhaps no place in the current manuscript. As stated in the reply to the previous point, we have removed the discussion of the implications of our current study to eukaryogenesis from the revised manuscript.

Having said that, I am happy that R3 has picked up exactly the same major concerns as I did with respect to e.g. the phrasing on mito (gene) loss and the BUSCO controversy.

We appreciate these comments and hopefully have resolved the concern in the previous answers.

Reviewer #2 (Significance):

Using draft genome sequencing of the free-living Paratrimastix pyriformis and the sister lineage oxymonad Blattamonas nauphoetae, Novack et al. infer the metabolic potential of the two protists using comparative genomics. The authors conclude that the common oxymonad ancestor lost the mitochondrion/mitosome and discuss general strategies for adapting to commensal/symbiotic life-style employed by this taxon. Some elaborations on pathways go on for several paragraphs and feel unnecessarily stretched, which made those sections of the paper rather difficult to digest. This might be also be because the work, and all conclusions drawn, depend entirely on incomplete (ca. 70-80%) genome data and simple similarity searches, and e.g. no kind of biochemistry or imaging is presented to underpin the manuscripts discussion.

We have addressed the concern about the possible incompleteness of our genome data above, demonstrating it is not substantiated ad stems from an inadequate interpretation of quality measures we provide in the manuscript. We hope that the revised manuscript, which is streamlined and more concise compared to the initial submission, conveys the key messages in a substantially more persuasive way and will be appreciated by a broad community of readers.

Reviewer #3 (Evidence, reproducibility and clarity):

Summary:
The genome sequences of two members of the protist group Preaxostyla are presented in this manuscript: Paratrimastix pyriformis and Blattamonas nauphoetae. The authors use a comparative genomics and phylogenetic approaches and compare the new genome datasets with three previously available genomes and transcriptomes from the group. The availability of genome-scale data from five Preaxostyla species is powerful to address interesting basic evolutionary questions. A substantial part of the manuscript is spent on testing the hypothesis of

mitochondrial loss in the oxymonad lineage, which turns out to be supported. The datasets are also explored regarding the role of lateral gene transfer in the group, metabolic diversification and the evolution of Golgi.

Major comments:
I find the manuscript very interesting with many different fascinating results presented. However, the manuscript is very long. Two genome sequences are presented and it is not clear to me what the main question was when this project was initiated and why these two species was selected to answer this question. I do not see an obvious reason for sequencing the P. pyriformis genome if the mitochondrial loss was the main question (given that a transcriptome was already available). Why not spend the time and resources on a member of Preoxystyla, which lacked previous data? The authors should more clearly state why these organisms were chosen to answer the main question or questions of the study.

We are sorry for having done a poor job when explaining the choice of the taxa for the comparison. The idea was to sample an outgroup of oxymonads (*P. pyriformis*) and a representative of other clades of oxymonads than *M. exilis* (*B. nauphoetae* and *S. strix*) for which it was feasible to obtain the data, or the data were already available. Obviously, more representatives of morphologically a probably also genetically diverse oxymonads should be investigated (e.g. *Pyrsonympha, Oxymonas, Saccinobacullus*) and we have such a plan but these organisms are difficult to work with. We considered it necessary to sequence the genome of *P. pyriformis*, and not rely on the transcriptome only, to avoid the issue of data set incompleteness (raised also by R2). Transcriptomes by nature provide an incomplete coverage of the full gene complement of the species, while our genome assemblies are close to complete, as we explain elsewhere.

The evolution of MROs have received substantial attention from the protist research community since the 1990's. During this period the mitochondrial organelle have been considered essential for eukaryotes. Therefore, the result presented in the manuscript has a high significance. However, I am not convinced that it is appropriate to use the term "evolutionary transition" for the mitochondrial loss. The loss of MRO is the endpoint of a gradual change of the internal organisation of the cell that probably started when the ancestor of these organism adapted to an anaerobic lifestyle. The last step described in the manuscript probably had little impact on how these organisms interacted with their environment. The presence or absence of biosynthesis of p-cresol by some, but not all, Preaxystyla probably is much more significant from an ecological point of view. My point is that the authors need to consider how they use the term evolutionary transition and be explicit about that.

We appreciate the comment concerning the use of the term “evolutionary transition”. Nevertheless, we believe there is no real consensus in the literature on what is and what is not an “evolutionary transition”, and the application of the term to specific cases is more or less arbitrary. For a lack of a standardized or better terminology, we have kept the term to refer to three evolutionary changes in the evolution of the Preaxostyla lineage that are particularly

important from the cytological or ecological perspective, i.e. dispensing with the mitochondrion, reorganizing the Golgi apparatus by losing the stacked arrangement of the cisternae, and gaining the endobiotic life style.

In the abstract the main finding is describes as "the data confirm the complete loss of mitochondria and every protein that has ever participated in the mitochondrion function for all three oxymonad species (M. exilis, B. nauphoetae, and Streblomastix strix) extending the amitochondriate status to the whole Oxymonadida.". I find this a really interesting observation, but I do find the wording a bit too bold for several reasons:
• Not every protein that has participated in the mitochondrial function is known.
• Mitochondrial proteins could be present in oxymonads, but divergent beyond the detection limit for existing methods.
• Genes for one or several mitochondrial proteins could be present in one or more oxymonad genomes, but remain undetected due to the incomplete nature of the datasets.

Although I do think that the authors' claim very well could be true, I don't think their data fully support it. Therefore, it needs to be rephrased.

As a result of our decision to streamline the manuscript by removing the final part of Results and Discussion (“No evidence for subcellular retargeting of ancestral mitochondrial proteins in oxymonads”, the revised manuscript no longer support the statement “the data confirm the complete loss of … every protein that has ever participated in the mitochondrion function for all three oxymonad species” that is criticized by the reviewer, and hence the statement has been removed from the abstract. This addresses bullet point 1. As for bullet points 2 and 3, the proof of absence is in principle impossible to deliver, and we have been fighting with this already in the Karnkowska et al. 2016 paper. Although our certainty will never reach 100% (this is in fact impossible for a scientific, i.e., falsifiable, hypothesis), the mounting of evidence through studies gives the hypothesis on the amitochodriate status of oxymonads more and more credit. The genes for mitochondrial marker proteins have not been detected by the most sensitive methods available neither in the first genome assembly of *M. exilis* (Karnkowska et al. 2016), nor in the improved *M. exilis* genome assembly composed of only 101 contigs (Treitli et al. 2021), nor in either of the other two oxymonad species investigated here. On the other hand, they were readily detected in the data sets of *P. pyriformis* and *T. marina*. What is the probability that these genes always hide in the assembly gaps, or that they have all escaped recognition? Obviously, this probability is not zero, but we believe it is approaching so low values that it is reasonably safe to make the conclusion on the amitochondriate status of these species.

The sentence was changed to: "*Our results provide insights into the metabolic and endomembrane evolution, but most strikingly the data confirm the complete loss of mitochondria for all three oxymonad species investigated (M. exilis, B. nauphoetae, and Streblomastix strix), suggesting the amitochondriate status may be common to Oxymonadida.*"

The third point maybe could be analysed further. BUSCO scores are reported, but also argued

not being reliable for this group of organisms (which is true). Would it, for example, be useful to analyse how large fraction of the BUSCO proteins found in all non-Preoxystyla metamonada genomes that are present in the various Preoxystyla datasets?

We provide a comprehensive answer to a similar comment of reviewer 2 above (page 6-8). We performed the requested analysis and provide the result in Supplementary file 11. In this table, we record presence/absence of each gene from the BUSCO set for our data sets and the highly complete "standard" datasets of *Trypanosoma brucei*, *Giardia intestinalis* and *Trichomonas vaginalis.* Of the 303 genes, 117 were present in all data sets and 17 in none (see column I). 20 were present only in *Trypanosoma* and not in metamonads. 6 were present in all Preaxostyla and absent in other metamonads (*Trichomonas* and *Giardia*), 44 were present in all Preaxostyla and *Trichomonas* and absent in *Giardia*, suggesting high divergence of this species. Only 23 (marked by *) were present in the three "standard" genomes and absent in one or more Preaxostyla species. Of those 8 and 8 were absent specifically in *S. strix* and *P. pyriformis*, respectively, but only 1 was absent specifically in *M. exilis* and no such case was observed in *B. nauphoetae.* We conclude that this non-random pattern argues for lineage-specific divergence rather than incomplete data sets, particularly in the case of *M. exilis* and *B. nauphoetae.*

Line 160-161: 15 LGT events specific for the Preaxostyla+Fornicata clade is reported. This is an exciting finding because it supports a phylogenetic relationship between these two groups. But such an argument is only valid if the observed pattern is more common than the alternative hypotheses (Preaxostyla+Parabasalids and Fornicata+Parabasalids). How many LGT events support each of these groupings? How are these observation affected by the current taxon sampling with the highest number of datasets from Fornicata? How were putative metamonada-to-metamonada LGTs treated in this context?

19 LGT are uniquely shared between Preaxostyla+Parabasalids, which is more than the number of shared LGTs between Preaxostyla and Fornicata. No common LGT was unique to Fornicata+Parabasalids. However, the latter is a direct consequence of our investigation method, which involved reconstruction phylogenies of genes present in Preaxostyla, and not across all metamonads. So, we do not have a way to investigate LGT gene families uniquely shared between Fornicata and parabasalids.

When it comes to the effect of taxon sampling, we agree that it is possible that the number of genes of horizontal origin shared between parabasalids and Preaxostyla is underestimated because of the lower taxon sampling in parabasalids. However, it is still larger (19) than the number of LGTs shared uniquely between fornicate and Preaxostyla (15). In addition, while the taxon sampling is larger in fornicates, it also contains some representatives of closely related lineages (e.g., *Chilomastix caulleryi* and *Chilomastix cuspidate*) which, while they increase the number of fornicate representatives, does not increase the detection of shared genes between fornicates and Preaxostyla. Altogether, it's difficult to estimate how the current taxon sampling is biasing the detection of LGTs one way or another.

Review COMMONS

Regarding metamonad-to-metamonad putative LGTs: we did not consider this possibility for the sake of not overestimating the number of gene transfers for two main reasons. First of all, our LGT detection relies on the incongruence between species tree and gene tree. The closer the lineages are in the species tree, the more difficult it is to interpret any incongruence in the gene tree as single protein phylogenies are notoriously poorly resolved because they rely on the little phylogenetic signal contained in few amino-acid positions. Because of this, small incongruences with the species tree could either reflect recent LGT events between metamonads, or simply blurry phylogenetic signal. Second, we can certainly use the argument that a limited taxonomic distribution among metamonads favors an LGT event between them. However, here again, the closer the lineages involved are, the more difficult it is to distinguish a scenario where one lineage was the recipient of an LGT from prokaryote before donating it to another metamonad, from a scenario involving a single ancestral LGT from prokaryotes to metamonads, followed by differential loss, leading to a patchy taxonomic distribution. Finally, we are working with both limited taxon sampling and incomplete genomic/transcriptomic data, which makes it more difficult to identify true absences. For all these reasons, we chose to be conservative and invoke the smallest number of LGT events.

The authors have used a large-scale approach to make single-gene trees for inferences of LGT. In other parts of the manuscript inferences of evolutionary origins of single genes are made without support of phylogenetic trees. I find this inconsistent and argue that the hypothesis of the origin of a specific protein should be tested with the same rigor whether it is a putative LGT, gene duplication, gene loss or an ancestral member of LECA. Specific cases where I think a phylogenetic analysis is needed includes:

- Line 222-223: It is concluded that Rsg1 is a component of LECA.
- Line 307: HgcAB are argued to be acquired by LGT of a whole opeon.
- Lines 350-355: It is unclear how the different numbers of transporters are interpreted (loss or expansion by duplication). This could be address with phylogenetics.
- Lines 407-408: A tree should support the claim of LGT origin. (PFP)
- Lines 414-415: The different origins of glycolytic enzymes should be supported by data or references.
- Line 486: Trees or a reference (if available) should support the claim for LGT.

As requested, trees were constructed for HgcA, HgcB, PFP and the transporters AAAP, CTL, ENT, pATPase, and SP. Citations were added for the glycolytic enzymes and the ADI pathway. No tree for Rsg1 is needed, as this is a eukaryote-specific protein lacking any close prokaryotic relatives. The inference on its presence in the LECA is based on the phylogenetically wide, however patchy, distribution across the eukaryote phylogeny. Testing possible eukaryote-eukaryote LGTs is hampered by a limited phylogenetic signal in the short and rapidly evolving Rsg1 sequences, resulting in very poorly resolved relationships among Rgs1 sequence in a tree we attempted to make (data not shown). For this reason, we opt for not presenting any phylogenetic analysis for Rsg1.

Review COMMONS

Lines 530-531 and 773-774: "The switch to the SUF pathway in these species has apparently not affected the number of Fe-S-containing proteins but led to a decrease in the usage of 2Fe-2S clusters." I find it difficult to evaluate if the data support this because no exact numbers or identities are given for 2Fe-2S and 4Fe-4S proteins in the various genomes in Suppl. Fig. S4 or Supplementary file 4.

The functional annotation of all detected FeS clusters containing proteins is provided in Supplementary Table S8 including the types of predicted clusters (columns G or F). Basically, the only putative 2Fe2S cluster containing proteins in species of oxymonad is xanthine dehydrogenase, while *Paratrimastix* and *Trimastix* contain also 2Fe2S cluster-containing ferredoxins and hydrogenases.

The method used in the paper varies between the different parts of the paper. One example is single gene phylogenies, which are described three times in the method section [Lines 959-973, lines 1011-1034, lines 1093-1101], in addition to the automated approach within the LGT detection pipeline lines 923-926]. The approaches are slightly different with, for example, different procedures for trimming. This makes it difficult to know how the different presented analyses were done in detail. No rationale for using different approaches is given. At the least, it should be clear in the method section which approach was used for which analysis.

The reviewer is correct, and we apologize for the inconsistency. The reason is only historical – the analyses were performed by different laboratories in different periods of time. We believe this fact does not make our results less robust, although it does not "look" nice and makes the description of the methods employed longer. We have double-checked the description and introduced slight changes as to make it maximally clear which method has been used for particular analyses presented in the Results and Discussion.

Specific comments on single gene phylogenies:

• Line 966-967: Why max 10 target sequences?

The limit of 10 was applied in order to keep the datasets in manageable dimensions. The sentence has been changed to: *" In order to detect potential LGT from prokaryotes while keeping the number of included sequences manageable, prokaryotic homologues were gathered by a BLASTp search with each eukaryotic sequence against the NCBI nr database with an e-value cutoff of 10-10 and max. 10 target sequences.*"

• Lines 996-998: Is it a problem that these are rather old datasets?

Although the publications are slightly older the set of queries is absolutely sufficient for the purpose.

Minor comments:

I appreciate that many data is included as supplementary material. However, the organisation of the data could be improved. The numbering of the files is not included in their names or within the files, as far as I could find. Descriptions of the files are often missing and information on the annotation such as colour coding is not always included. These aspects of the supplementary material needs to be strengthened in order to make it more useful. Specific comments:
• Supplementary file 1, Table 1: accession numbers are missing. Kipferlia bialta appears to have a much smaller number of sequences than reported in the publication. The file consists of three tables and it would be very helpful if the reference in the main manuscript indicate the table number.
• Supplementary file 4: The trees lack proper species names and a documented colour coding. There are multiple trees in the file, which make it difficult to find the correct tree. I would appreciate if the different trees were labelled A, B, C, etc., and if these were used in the main text.

Supplementary file 1: Accession numbers were added.

Supplementary file 4: Species names and alphabetical labelling were added. Colour coding was explained in the text at the first mention of the file: "(Supplementary file 4 H; Preaxostyla sequences in red)."

o There is no HPAD-AE tree (as indicated on line 258), but a HPAD tree. Which part of the tree contain the described fusion protein?

Thank you for spotting the mistake. There should have been “HPAD” instead of “HPAD-AE” indicated in the text. The sentence has been changed to:" The *P. pyriformis* HPAD sequence is closely related to its homolog in the free-living archamoebid *M. balamuthi* (Supplementary file 4 K), the only eukaryote reported so far to be able to produce p-cresol (Nývltová et al. 2017)."

o Line 280-281: "UbiE homologs occur also in some additional metamonads, including the oxymonad B. nauphoetae and certain fornicates." These sequences should be clearly highlighted in the tree.

We discovered these additional UbiE homologs only after the tree presented in the supplement had been constructed, so these sequences are missing from it. To ensure consistency we have decided to remove the remark on the presence of UbiE homologs metamonads other than *P. pyriformis*, so it is no longer part of the revised manuscript.

o Lines 538-544: A three-gene system is mentioned, but only two AmmoMemoRadiSam trees are found.

This part has been removed while streamlining the manuscript.

• Supplementary file 6: I find it difficult to find the proteins discussed in the text, for example "the biosynthesis of p-cresol from tyrosine (line 254-255)".

Abbreviations identifying the different enzymes have now been added to all mentions in the text, facilitating their localization in the supplementary file: "*P. pyriformis encodes a complete pathway required for the biosynthesis of p-cresol from tyrosine (Supplementary file 6), only the second reported eukaryote with such capability. This pathway consists of three steps of the Ehrlich pathway (Hazelwood et al. 2008) converting tyrosine to 4-hydroxyphenyl-acetate (AAT, HPPD, ALDH) and the final step catalyzed by a fusion protein comprised of 4-hydroxyphenylacetate decarboxylase (HPAD) and its activating enzyme (HPAD-AE).*"

• Supplementary file 11: Which group of species are highlighted in red? How do I know from which species these sequences are (I can make educated guesses, but prefer full species names). I do not find any reference to this file in the main manuscript.

We apologise for this inconvenience. The taxon labels in the treed in this supplementary file have been corrected to contain full species names.

Line 227-228: "630 OGs seem to be oxymonad-specific or divergent, without close BLAST hits". It is unclear if BLAST searches includes only a representative of each 630 OGs, or every single protein in these OGs.

The BLAST searches include every single protein in the investigated OGs. We clarified it in the text: "*Of these, 630 OGs seem to be oxymonad novelties or divergent ancestral genes, without close BLAST hits (e-value < 10^{-15}) to any of these sequences.*"

Line 243: I think it is five LGT mapped to internal nodes of Preoxystyla in Figure 1 (1+3+1).

You are correct, we apologize for the mistake. The sentence has been changed to: "*Also, 46 LGT events were mapped to the terminal branches and 5 to internal nodes of Preaxostyla, suggesting that the acquisition of genes is an ongoing phenomenon, and it might be adaptive to particular lifestyles of the species.*"

Lines 325-331: The argument would be stronger with a figure showing the fusion and the alignment indicating the conserved amino acids mentioned in the text.

We agree with the reviewer but for the sake of space, we finally decided not to include a new figure.

Review COMMONS

Lines 425: "none of the species encoded" should be replaced by something like "none of the enzyme could be detected in any of the species" (the datasets are incomplete).

The sentence has been changed to: "*None of the alternative enzymes mediating the conversion of pyruvate to acetyl-CoA, pyruvate:NADP+ oxidoreductase (PNO) and pyruvate formate lyase (PFL), could be detected in any of the studied species."*

Line 455: "suggesting a cytosolic localization of these enzymes in Preaxostyla." The absence of a phylogenetic affiliation with the S. salmonicida homolog does not preclude a MRO localisation.

The sentence was changed to: "*Phylogenetic analysis of Preaxostyla ACSs (Supplementary file 4 B) shows four unrelated clades, none in close relationship to the* S. salmonicida *MRO homolog, consistent with our assumption that these enzymes are cytosolic in Preaxostyla.*"

Lines 570-571: "Manual verification indicated that all the candidates recovered in oxymonad data sets are false positives" Using which criteria?

The manual verification was based on the annotation of predicted proteins by BLAST and InterProScan. If the annotations did not correspond to the suggested function, they were considered false positives. For example, the protein BLNAU_15573 of *Blattamonas nauphoetae* was detected by Sam50 HMM profile and thus was considered a candidate for Sam50 proteins. Its functional annotation from BLAST was, however, unrelated to Sam50 ("putative phospholipase B"). Therefore, this candidate was concluded as a false positive hit of the HMM search resulting from the very high sensitivity of this method.

We clarified this in the Results

"*Reciprocal BLASTs indicated that all the candidates recovered in oxymonad data sets are very likely to be false positives based on the annotations of their top BLAST hits (mainly vaguely annotated kinases, peptidases and chaperones) (Fig. 6, Supplementary file 9).*".

And Material and Methods

"*Any hits received by the methods described above were considered candidates and were furter inspected as follows. All candidates were BLAST-searched against NCBI-nr and the best hits with the descriptions not including the terms 'low quality protein', 'hypothetical', 'unknown', etc. were kept. For each hit, the Gene Ontology categories were assigned using InterProScan-5.36-75.0. If the annotations received from BLAST or InterProScan corresponded to the originally suggested function, the candidates were considered as verified. Otherwise, they were considered as false positives.*"

Lines 743-755: "Similar observations were made in other protists with highly reduced mitochondria, such as G. intestinalis or E. histolytica,..." References are needed.

This part of the manuscript has been removed while streamlining the text.

Line 849: How was the manually curation done for the gene models in the training set?

The sentence has been changed to: "For de novo prediction of genes, Augustus was first re-trained using a set of gene models manually curated with regard to mapped transcriptomic sequences and homology with known protein-coding genes."

Lines 853-856: It is a bit unclear which dataset was used for BUSCO and downstream analysis. Was it the Augustus-predicted proteins, or the EVM polished?

The sentence has been changed to: "The genome completeness for each genome was estimated using BUSCO v3 with the Eukaryota odb9 dataset and the genome completeness was estimated on the sets of EVM-polished protein sequences as the input."

Lines 858: What is it meant that KEGG and similarity searches was used in parallel (what if both gave a functional annotation?)?

A sentence has been added for clarity: "KEGG annotations were given priority in cases of conflict."

Lines 861-862 and 1007-1008: Which genes or sub-projects does this apply to? How many genes were detected in this procedure?

The sentence has been changed to make this clear: "*Targeted analyses of genes and gene families of specific interest were performed by manual searches of the predicted proteomes using BLASTp and HMMER (Eddy 2011), and complemented by tBLASTn searches of the genome and transcriptome assemblies to check for the presence of individual genes of interest that were potentially missed in the predicted protein sets (single digits of cases per set). Gene models were manually refined for genes of interest when necessary and possible.*"

Lines 878-879: It is not clear to me why the sum of the two described numbers should be as high as possible and would appreciate an argument or a reference.

When optimizing the inflation parameter of OrthoMCL, we reasoned that the optimal level of grouping/splitting for our purpose should result in the highest number of orthogroups containing all representatives of the groups of interest (i.e. Preaxostyla) but no other species – pan-Preaxostyla orthogroups. When going down with the values, you observe more and more groupings of pan-Preaxostyla OGs with others (indication of overgrouping) in the opposite

direction you observe splitting of pan Preaxostyla OGs which indicates oversplitting. Because we were optimizing the inflation parameter for Preaxostyla and Oxymonadida at the same time, we maximized the sum of pan-Preaxostyla and pan-Oxymonadida groups.

Lines 879-881: "Proteins belonging to the thus defined OGs were automatically annotated using BLASTp searches against the NCBI nr protein database (Supplementary file 1)." Why were these annotated in a different way (compare lines 857-859).

This little inconsistency resulted from the fact that these parts of the analyses were performed by different researchers who did not cross-standardize the procedures. This inconsistency has no effect on the downstream analyses and conclusions as the annotations from Supplementary file 1 were not used in any further analyses.

Lines 894-957: "Detection of lateral gene transfer candidates":
• It is not clear which sequences were tested in the procedure. All Preaxostyla, or all metamonada? I think I am confused because in the result sections you only report numbers for Preaxostyla, but in the method section metamonada is mentioned repeatedly.

Thank you for noticing. There was indeed some inconsistency in our writing.
We did an all-against-all search using all metamonads. However, we filtered out all homologous families in which Preaxostyla were not present or that had no hit against GTDB. So in the end, the LGT search was restrained to protein families containing Preaxostyla homologues. We corrected the wording in our method section.

• It would be easier to follow the procedure if numbers are provided for the different steps.

We are not sure what numbers the reviewer refers to here.

• Why was only small oxymonad proteins discarded (line 900)?

This is indeed a mistake. We meant “Preaxostyla proteins”. This is because we only considered Preaxostyla sequences with significant hits against GTDB as a starting point, so we aimed to first remove those that might be too short to yield reliable phylogenies.

• Line 911: How many sequences were collected?

Up to 10,000 hits were retained. We have added that information to the text.

• Lines 916-919: What is the difference between the protein superfamilies (line 916) and the

Review COMMONS

OGs (line 919)? Are the OGs the same orthogroups that is described earlier in the method section? How are the redundancy of NCBI nr entries retrieved in different searches dealt with?

We understand the confusion here. It primarily stemmed from two different ways to establish homologous families across the manuscript because of different researchers being responsible for different parts. Protein superfamilies that were used for reconstructing the single protein trees used for the LGT analyses were assembled based on the procedure describe line 916-919 ("Protein superfamilies were assembled by first running DIAMOND searches of all metamonad sequences against all (-e 1e-20 --id 25 --query-cover 50 --subject-cover 50). Reciprocal hits were gathered into a single FASTA file, as well as their NCBI nr homologues."). However, this was a somewhat stricter procedure than the one used to establish the OGs that are discussed in the rest of the manuscript (because of the e-value and identity cut-off used), so we eventually enriched the datasets with the putatively missing metamonad sequences that were present in the OGs but not in the initial superfamily assembly. However, since these were often more divergent sequences, we did not use these as queries for our BLAST searches against prokaryotes.

Line 987-989: "...was facilitated by Rsg1 being rather divergent from other Ras superfamily members" This statement is vague. What does it mean in practise?

The sentence has been changed to: " The discrimination was facilitated by Rsg1 having low sequence similarity to other Ras superfamily members (such as Rab GTPases)."

Lines 1037-1038: Why were these proteins re-annotated?

They were not. We are sorry for this mistake, which has been fixed in the revised manuscript.

Figures: The figures would be easier to follow if the colour coding for the five different species were consistent between the figures.

This is a good point, the colour coding has been unified across all figures.

Figure 1: It appears that the Venn diagram in C only shows the Preaxostyla-specific protein in B, not all OGs for which contain Preaxostyla proteins. This is not clear from legend or from the figure itself. The same comment applies to D.

The interpretation of the figure by the reviewer is correct; we have modified the legend to make the meaning of the figure easier to understand.

Figures 2 and 6: It would be clearer with panel labels A, B, etc, instead of "upper" and "lower" panel, as in the other figures.

This is a fair point, we have added the alphabetical labels proposed by the reviewer to the figures.

Figure 6: What is the colour code in the figure? The numbers within the boxes are not aligned.

We have added an explanation of the color code to the legend and edited the figure to make it aesthetically more pleasing.

Supplementary figures 1-3: What do green and magenta indicate in the figure?

As with the previous figure, the color code is now explained in the revised legend.

** Referees cross-commenting**
I agree with the other reviewers that the discussion of the functional and ecological implications of the LGTs could be developed.

We understand the reviewers but as already explained in response to Reviewer 1, we have decided not to extend the already rather long manuscript further. We believe that the several exemplar LGT cases that we do discuss in detail provide a good impression of the significance of LGT in the evolution of Preaxostyla.

In contrast to reviewer 2, I do not see that the authors discuss their result in the context of eukaryogenesis in this manuscript. Maybe the reference reviewer 2 mention could be cited in the introduction together with Hampl et al. 2018 to acknowledge that there are different views about the importance of secondarily amitochondrial eukaryotes on our thinking about the origin of eukaryotes. I disagree with reviewer 2's objection against the wording "... and undergo pronounced morphological evolution" because I think Fig. 4 in Hampl 2017 shows a large morphological diversity among oxymonads.

We are glad to see that our perspective is not shared by other colleagues in the field. Nevertheless, having carefully considered the case we have decided to remove any mentions of eukaryogenesis from the revised manuscript, as we admit this topic is peripheral to the key message of our present study. On the other hand, we appreciate very much the note by the reviewer on the large morphological diversity among oxymonads – we have now added a similar remark to the revised manuscript (the last sentence of Conclusions).
