# Supplementary material for: Genomics of Preaxostyla Flagellates Illuminates the Path Towards the Loss of Mitochondria
Source: PLoS Genet. 2023 Dec 7;19(12):e1011050. doi: 10.1371/journal.pgen.1011050 (PMC10703272; doi:10.1371/journal.pgen.1011050)
Supplement: Attachment — Submitted filename: Review_Novak et al..pdf [file pgen.1011050.s015.pdf]

In this manuscript, Novák *et al.* provide genomic and transcriptomic analyses of two Preaxostyla representatives, namely *Paratrimastrix pyriformis* and *Blattamonas nauphoetae* classified in the group of Oxymonadida. Their analysis reveals unusual predicted metabolic capacity for *P. pyriformis* and the lack of mitochondrial protein hallmark in the predicted proteomes of both protists. The latter suggests that like oxymonad *Monocercomonoides exilis*, the first reported eukaryote with no mitochondria, *P. pyriformis* and *B. nauphoetae* are also amitochondriate organisms. The paper brings interesting evolutionary perspectives on eukaryotes and contributes to change our view of the mitochondria as clear defining feature of a eukaryotic cell.

1) As such the paper is very densely written and not easily accessible for the broader readership of PLOS Genetics. We suggest that the authors rewrite the presentation of their key findings revealed from their analysis and provide more evolutionary context so that the significance is more obvious.

2) The use of "ancestral features" in reference to the unique metabolic capacities of *P. pyrifromis* in the abstract is unclear. Is it meant for bacterial feature? If the *p*-cresol synthesis is indeed a bacterial feature, selenium volatilization is present in many aquatic plants. Maybe the authors can clarify what is an ancestral feature?

3) The authors mention in the abstract that their analysis provides insight into the evolutionary changes towards amitochondriality, endobiosis and unstacked Golgi. It is not obvious how these would be connected (or if they are at all) as evolutionary traits in the eukaryotic lineage. The title also indicates that the work highlights a possible evolutionary scenario for the loss of mitochondria in the eukaryotic lineage, but this scenario is not apparent to us from the analysis provided in this work. Maybe a short discussion addressing these points could be included in the manuscript.

4) Is the membrane transporter complement always a trait of an endobiotic lifestyle? Or are there known examples of free-living organisms with many transporters? Can the authors elaborate on this?

5) If the Golgi is currently viewed as a feature of eukaryotes, the extent and nature of the complexity of this organelle in the ancestor of eukaryotes remains unclear. Perhaps the authors can provide some context about this in their section on the evidence of a Golgi apparatus. For instance, it is unclear to us why the cisternal adhesion model is a key finding from the authors' analysis. Is this because such a finding anchors the two protists in the eukaryotic lineage despite the apparent complete loss of mitochondria? Are there different types of Golgi apparatus among eukaryotes?

6) We were curious about the heme prosthetic group in the predicted proteomes of the two protists. Is there any evidence for hemoproteins and heme synthesis enzymes in *P. pyriformis* and *B. nauphoetae*?

7) Is there evidence of peroxisomal proteins in the predicted proteomes of *P. pyriformis* and *B. nauphoetae*? This might be relevant information to include in this work considering peroxisomes are assumed to be present in almost all eukaryotes.
