# Supplementary material for: Genomics of Preaxostyla Flagellates Illuminates the Path Towards the Loss of Mitochondria
Source: PLoS Genet. 2023 Dec 7;19(12):e1011050. doi: 10.1371/journal.pgen.1011050 (PMC10703272; doi:10.1371/journal.pgen.1011050)
Supplement: Attachment — Submitted filename: Novak_Preaxostyla_Answers_20231021.pdf [file pgen.1011050.s016.pdf]

Answers to reviewers' comments

Reviewer #1:

Novák and colleagues present and excellent study - rich in detail and meticulously carried out. I agree with the previous reviewers that there is a lot in this paper -- but this is a clear strength (except for that it took me quite some time to digest it all)! Since this is already a revision, I will not add additional minor request here and there, but rather get to the point: the authors have done a great job of putting together a detailed comparative genomic investigation that not only counts some LGTs, but provides rich insights into exciting biochemistry of these fascinating organisms. Therefore I will refrain from making any other suggestions -- but one: please add a cladogram to Figure 1 to highlight for the general reader exactly *where* on the tree of eukaryotes we are (most readers of PLOS Genetics will not know what metamonads are)

Thank you for the positive assessment and a valuable suggestion. The manuscript was indeed missing an explanation of a broader phylogenetic context. Although the suggestion regards a figure that is no longer included in the revised manuscript, we still consider it valid. Therefore, we have included a schematic tree illustrating the phylogenetic position of Metamonada and the internal topology of Preaxostyla in the current Figure 1.

Reviewer #2:

In this manuscript, Novák *et al.* provide genomic and transcriptomic analyses of two Preaxostyla representatives, namely *Paratrimastrix pyriformis* and *Blattamonas nauphoetae* classified in the group of Oxymonadida. Their analysis reveals unusual predicted metabolic capacity for *P. pyriformis* and the lack of mitochondrial protein hallmark in the predicted proteomes of both protists. The latter suggests that like oxymonad *Monocercomonoides exilis*, the first reported eukaryote with no mitochondria, *P. pyriformis* and *B. nauphoetae* are also amitochondriate organisms. The paper brings interesting evolutionary perspectives on eukaryotes and contributes to change our view of the mitochondria as clear defining feature of a eukaryotic cell.

1) As such the paper is very densely written and not easily accessible for the broader readership of PLOS Genetics. We suggest that the authors rewrite the presentation of their key findings revealed from their analysis and provide more evolutionary context so that the significance is more obvious.

We have followed your comment, which also matched that of the third reviewer and the editor, and reorganised the manuscript. Firstly, we have removed the parts on comparative analyses of gene inventories, lateral gene transfers and the chapter on the metabolic peculiarities of *Paratrimastix pyriformis*. Furthermore, we have moved the topic of the mitochondrion disappearance in oxymonads to the beginning of Results and Discussion to reflect the fact that this is the major result of the entire study. We have also done our best to improve the text flow and we believe the manuscript has improved in this respect.

2) The use of “ancestral features” in reference to the unique metabolic capacities of *P. pyrifromis* in the abstract is unclear. Is it meant for bacterial feature? If the *p*-cresol synthesis is indeed a bacterial feature, selenium volatilization is present in many aquatic plants. Maybe the authors can clarify what is an ancestral feature?

This was indeed a confusing wording. It should not mean ancestral bacterial features. In any case, the part of the text you refer to has been removed during the shortening of the manuscript so this confusing issue is no longer present.

3) The authors mention in the abstract that their analysis provides insight into the evolutionary changes towards amitochondriality, endobiosis and unstacked Golgi. It is not obvious how these would be connected (or if they are at all) as evolutionary traits in the eukaryotic lineage. The title also indicates that the work highlights a possible evolutionary scenario for the loss of mitochondria in the eukaryotic lineage, but this scenario is not apparent to us from the analysis provided in this work. Maybe a short discussion addressing these points could be included in the manuscript.

These are valid concerns about the congruence between the title, the abstract, the body of the manuscript and the overall logic of the presented story. We do not think that the three transitions are directly related, however, they coincided in time and may be linked indirectly. To address the comment and to clarify our view, we have included a brief discussion before we proceed to elaborate on the transition to endobiosis:

"The collected -omic data set from Preaxostyla allows us to examine two other evolutionary changes besides the loss of the mitochondrion, the transition from a free-living to an endobiotic lifestyle and the loss of stacked Golgi. Although all three events occurred on the same branch of the phylogenetic tree, leading to the common ancestor of the three analyzed oxymonads, we have no clear evidence that they are directly related. However, an indirect link between them is possible. For example, the transition to an endobiotic mode of life could cause the loss of the methionine cycle in oxymonads. This change might subsequently allow for the loss of mitochondria, whose only essential role in free-living Preaxostyla species is to provide methyl groups for this cytosolic cycle."

4) Is the membrane transporter complement always a trait of an endobiotic lifestyle? Or are there known examples of free-living organisms with many transporters? Can the authors elaborate on this?

It is difficult to discuss this topic in general terms because there is very little previous research available. However, we agree that providing more context is desirable. We have included a short discussion comparing this situation to ion transporters' duplications at the transition to halophily in other organisms:

"Indeed, gene duplication in specific types of transmembrane transporters may be a common adaptation at the transition to a new environment in eukaryotes; for example, expansion in gene families encoding ion transporters has been observed in halophilic organisms [80–82] when compared to their mesophilic relatives."

5) If the Golgi is currently viewed as a feature of eukaryotes, the extent and nature of the complexity of this organelle in the ancestor of eukaryotes remains unclear. Perhaps the authors can provide some context about this in their section on the evidence of a Golgi apparatus. For instance, it is unclear to us why the cisternal adhesion model is a key finding from the authors' analysis. Is this because such a finding anchors the two protists in the eukaryotic lineage despite the apparent complete loss of mitochondria? Are there different types of Golgi apparatus among eukaryotes?

We thank the reviewer for their interest in this aspect of the paper. We have provided more background on the diversity of Golgi morphologies in eukaryotes, and more text to provide context for the results:

„Golgi bodies with non-canonical morphologies are relatively rare in eukaryotes, as compared with the hallmark stacked cisternal form [84]. However, such organelles are well-documented [84]. Surprisingly, the molecular basis for the canonical eukaryotic stacked morphology is unclear. Many proteins have been implicated in Golgi stacking, though none yet found to be universal. At the same time the cisternal adhesion model [85], an emerging but still relatively controversial theory, proposes that a sufficient amount of any one of several putative stacking factors could provide the adhesive property to keep the stacks together. A 2018 study by Barlow et. al. [86], looked for all proposed Golgi-stacking factors at the time by sampling genomic data from across eukaryotes possessing stacked and unstacked Golgi morphologies. No protein was found in a pattern that supported it as a necessary and sufficient for Golgi stacking, although the complement of Golgins inferred to be present in LECA did support the functional distinction of cis vs trans-Golgi. This finding was most consistent with the cisternal adhesion model. Nonetheless, the organisms sampled with divergent Golgi morphologies were still quite distantly related and thus the evidence supporting the model was still relatively weak."

6) We were curious about the heme prosthetic group in the predicted proteomes of the two protists. Is there any evidence for hemoproteins and heme synthesis enzymes in *P. pyriformis* and *B. nauphoetae*?

Thank you for raising this interesting point, which is clearly connected to adaptation to anaerobiosis and reduction of mitochondria. We have performed the searches for heme synthesis enzymes and recovered no homologues. We conclude that this synthesis is not present, like in other metamonads investigated before. Unfortunately, we have not found a way to search for heme-containing proteins systematically and extensively on the genome-scale. If the reviewer knows a tool that is capable of this, we are happy to use it and add this information. Currently, we had no choice but to search through functional annotations of Preaxostyla proteins and perform ad hoc homology searches. This way, we have recovered in each investigated genome at least one cytochrome b5 domain-containing protein, which most likely binds heme (Supplementary file 1). This allows us to claim that most likely all Preaxostyla use heme-containing proteins and probably sequester the heme cofactor from their food. To report on the findings, we have included a new paragraph in the Results and Discussion:

"The modification of mitochondria for life in low-oxygen environments typically leads to the loss of the capacity for the synthesis of heme [78], a cofactor of many proteins involved in redox reactions, namely the complexes of the respiratory chain. Preaxostyla are no exception in this respect as no homologues of these enzymes were identified in their datasets. Still, cytochrome b5 domain-containing proteins were recovered in all Preaxostyla (S6 File) indicating that they use hemoproteins and likely sequester heme from the food."

7) Is there evidence of peroxisomal proteins in the predicted proteomes of *P. pyriformis* and *B. nauphoetae*? This might be relevant information to include in this work considering peroxisomes are assumed to be present in almost all eukaryotes.

This is another interesting topic, thank you. We have performed the suggested searches and detected a divergent Pex19 homologue in all species besides *S. strix*, and Pex3 homologue in *P. pyriformis*. We conclude that peroxisomes are absent from Preaxostyla and the Pex3/Pex19 system functions for the delivery of tail-anchored proteins into the outer membrane of the MRO in *P. pyriformis*, as suggested by Zítek et al. 2022. To report on these results, we have included a new paragraph in the Results and Discussion:

"Besides modifications or a complete loss of mitochondria, living in low-oxygen environments and/or endobiotically also typically impacts on peroxisomes. While in most anaerobic/microaerophilic species, including all previously investigated Metamonada, the peroxisomes are absent, both free-living and parasitic Archamoebae recently turned out to possess modified versions of this organelle [73–75], as

does Proteromonas lacertae, a stramenopile that possesses the most reduced but seemingly functional peroxisomal organelle yet reported [76]. Specific genetic markers for the presence of peroxisomes are peroxins, a set of proteins involved in peroxisome biogenesis [77]. Peroxins are absent in the genome of the oxymonad M. exilis with a single exception of a divergent homologue of Pex19 [8]. This protein acts as a soluble cytoplasmic receptor for peroxisomal tail-anchored (TA) membrane proteins, which are inserted into the peroxisomal membrane with the help of the Pex3 anchor. The absence of a discernible Pex3 homologue in M. exilis indicates that the Pex19 homologue is involved in another cellular process. To map the situation in other Preaxostyla, we searched all datasets for homologues of human peroxins (S8 File). Besides putative homologues of Pex19, which were recovered in all species besides S. strix, and a Pex3 homologue recovered in P. pyriformis, no other peroxins were detected. The Pex3/Pex19 pair in P. pyriformis was already reported by Zítek et al. [17], who provided proteomic evidence for Pex3 being localized in the MRO and hypothesized that the Pex3/Pex19 system is involved in the targeting of TA proteins into the outer MRO membrane. Altogether, the results of our searches do not indicate the presence of peroxisomes in Preaxostyla lineage. Thus, the apparent absence of Pex3 in oxymonads seems to be another trait linked to the loss of the mitochondrion in these organisms.“

Reviewer #3:

Novák and colleagues present here a very interesting and important manuscript that describes the genomes of two Preaxostyla flagelleates, Paratrimastix pyriformis and Blattamonas nauphoetae. The 2016 manuscript from this group describing the complete loss of a mitochondrial organelle from Monocercomonoides was monumental, and this manuscript goes on to show that at least one more oxymonad has apparently lost MROs, opening the possibility that all oxymonads are amitochondriate.

Overall, I congratulate the authors on the overall quality of the work presented in this manuscript. I do not have major concerns about the methods employed in making the most important conclusions of the manuscript. The completeness of the genomes (central to the arguments about mitochondria) is reasonably good (though minor comments later on), and the bioinformatics searches for LGTs, and Golgi/MRO proteins were rigorous and well thought out. It will be very interesting to learn, once additional species are investigated, whether all oxymonads are amitochondriate, or if Monocercomonoides and Blattamonas are just two instances of mitochondrial loss!

In contrast to my positive feelings on the quality of the work done here, I feel that too many - and somewhat disconnected - stories are being told. The net effect of this is that the impact of your biggest point (about MRO loss) is being blunted. I understand that it's hard to leave out interesting details of your analyses, but the manuscript is very long, and almost feels like it could be two separate stories (core proteomes/LGT vs MRO). I bring this up mainly because I feel that the main conclusions presented here really are important.

Upon revising the manuscript in light of the comments by the external reviewers and the editor, we admit that the manuscript tried to present too many topics and analyses that did not necessarily connect very organically. So thank you for your suggestion to split the manuscript into two, which was also recommended by the editor. We decided to remove from the manuscript the parts on comparative analyses of gene inventories, lateral gene transfers and the chapter on the metabolic peculiarities of *Paratrimastix pyriformis*, which we will publish independently in the future. We have also restructured the text to start with analyses of the presence/absence of mitochondria and its effect on selected pathways, which is the crucial message of our study. The new version of the manuscript then continues with a shorter passage focused on the transition to endobiosis and evolution of

unstacked Golgi in oxymonads. We hope the reduced complexity and the improved flow of the text now make our manuscript a more pleasant reading.

Some very minor points: Line 83 - while I'm enthusiastic about the results, I'm not sure that such a limited number of genomes can yet answer if oxymonads are amitochondriate 'in general'

This is a valid point. The simplest scenario drawn from our data places the loss of mitochondria in the common ancestor of *Monocercomonoides*, *Streblomastix* and *Blattamonas*. Unfortunately, we do not have a robust oxymonad phylogeny that would enable us to demonstrate how broad the diversity of oxymonads descending from this common ancestor is. The 18S rRNA tree of Treitli et al. (2018), indicates it is only a part of oxymonad diversity, however, the node of this ancestor is very deep and the bootstrap support for the backbone is very low. To avoid overstatements, we have down toned the conclusions as follows:

In Abstract

"…suggesting the amitochondriate status is common to a large part if not whole group of oxymonadida."

In Conclusions

"This shows that the absence of mitochondria is not a unique feature of a single species, M. exilis, but applies to a wider range of oxymonads, putatively the whole group."

Line ~100 (genome completeness analysis). I understand that protist genomes often do not give a near 100% score in BUSCO/CEGMA because many universal proteins aren't universal. One additional strategy could be looking for all cytosolic ribosomal proteins; these are highly conserved, typically not lost, and would be readily identifiable from transcriptome data if they aren't present in the genome, as they are highly expressed. Another thought might be looking to see what proportion of transcripts aren't represented in the genome assembly.

Thank you for the suggestions. We have searched the genomes for cytosolic ribosomal proteins and found 75 of 79 proteins in *P. pyriformis* and all 79 in *B. nauphoetae*. We also performed an additional independent assessment of genome completeness using Merqury, which is reference-free, returing the completeness values of 94.01% for *P. pyriformis* and 98.24% for *B. nauphoetae*. A paragraph describing these analyses have been included in the manuscript:

"To avoid a bias caused by evolutionary divergence, we also attempted to estimate genome completeness using reference-independent approaches with Merqury [19]. Using this method, we obtained a completeness of 94.01% and 98.24% for P. pyriformis and B. nauphoetae, respectively (Table 1). Finally, we also searched for 79 conserved ribosomal proteins [20]. We identified 74 of them in the predicted gene set of P. pyriformis, with the remaining five identified by manual searches in the genome (S1 File, Sheet 2). A comparable result was achieved for B. nauphoetae, where we identified 76 of the ribosomal proteins in the predicted gene set, and the remaining three were identified by a manual search in the genome (S1 File, Sheet 3). Altogether our analyses suggest that the P. pyriformis and B. nauphoetae genome drafts are highly complete."

Line ~146 - not a critique but a question - are there any consistent signals amongst these LGTs that would give hints about previous symbiotic partners?

This would indeed be very interesting. Sadly, we do not see any consistent signal or patterns in the single gene tree topologies that would provide such hints. Regardless, the section dealing with LGT has been removed from the revised manuscript.
